# In vivo site-specific engineering to reprogram T cells

**DOI:** 10.1038/s41586-026-10235-x

**Published:** 2026-03-18

**Authors:** William A. Nyberg, Pierre-Louis Bernard, Wayne Ngo, Charlotte H. Wang, Jonathan Ark, Allison Rothrock, Gina M. Borgo, Gabriella R. Kimmerly, Jae Hyung Jung, Vincent Allain, Jennifer R. Hamilton, Alisha Baldwin, Robert Stickels, Sarah Wyman, Safwaan H. Khan, Shanshan Lang, Donna Marsh, Niran Almudhfar, Catherine Novick, Yasaman Mortazavi, Shimin Zhang, Mahmoud M. AbdElwakil, Luis R. Sandoval, Sidney Hwang, Simon N. Chu, Hyuncheol Jung, Chang Liu, Devesh Sharma, Travis McCreary, Zhongmei Li, Ansuman T. Satpathy, Julia Carnevale, Rachel L. Rutishauser, M. Kyle Cromer, Kole T. Roybal, Stacie E. Dodgson, Jennifer A. Doudna, Aravind Asokan, Justin Eyquem

**Affiliations:** 1 Department of Medicine, Division of Hematology-Oncology, University of California, San Francisco, San Francisco, CA USA; 2 Gladstone-UCSF Institute of Genomic Immunology, San Francisco, CA USA; 3 Innovative Genomics Institute, University of California, Berkeley, Berkeley, CA USA; 4 California Institute for Quantitative Biosciences, University of California, University of California, Berkeley, Berkeley, CA USA; 5 Gladstone Institute of Data Science and Biotechnology, San Francisco, CA USA; 6 Department of Molecular Genetics & Microbiology, Duke University School of Medicine, Durham, NC USA; 7 Division of Experimental Medicine, Department of Medicine, University of California, San Francisco, San Francisco, CA USA; 8 Université Paris Cité, INSERM UMR1342, Hôpital Saint-Louis, Paris, France; 9 Department of Molecular and Cell Biology, University of California, Berkeley, Berkeley, CA USA; 10 Department of Microbiology and Immunology, University of California, San Francisco, San Francisco, CA USA; 11 Department of Surgery, Division of Pediatric Surgery, University of California, San Francisco, San Francisco, CA USA; 12 Parker Institute for Cancer Immunotherapy, San Francisco, CA USA; 13 UCSF Helen Diller Family Comprehensive Cancer Center, University of California, San Francisco, San Francisco, CA USA; 14 Institute for Human Genetics (IHG), University of California, San Francisco, San Francisco, CA USA; 15 Howard Hughes Medical Institute, University of California, Berkeley, Berkeley, CA USA; 16 Molecular Biophysics and Integrated Bioimaging Division, Lawrence Berkeley National Laboratory, Berkeley, CA USA; 17 Department of Chemistry, University of California, Berkeley, Berkeley, CA USA; 18 Department of Cellular and Molecular Pharmacology, University of California, San Francisco, San Francisco, CA USA; 19 Li Ka Shing Center for Genomic Engineering, University of California, Berkeley, Berkeley, CA USA; 20 Department of Surgery, Duke University School of Medicine, Durham, NC USA; 21 Department of Biomedical Engineering, Duke University, Durham, NC USA; 22 Present Address: Center for Hematology and Regenerative Medicine, Department of Medicine Huddinge, Karolinska Institutet, Huddinge, Sweden; 23 Present Address: Science for Life Laboratory, Department of Medicine Huddinge, Karolinska Institutet, Solna, Sweden; 24 Present Address: Azalea Therapeutics, Berkeley, CA USA

**Keywords:** Immunotherapy, Tissue engineering, Genetic engineering, Cancer immunotherapy

## Abstract

Engineered T cells, reprogrammed to express chimeric antigen receptors (CAR) or T cell receptors (TCR), have transformed cancer treatment and are being explored as therapeutics for autoimmune and infectious diseases. Enhancing T cell function through genome editing, either by disrupting endogenous genes or precisely inserting DNA payloads, has shown considerable promise^1^. However, the ex vivo manufacturing process is lengthy and costly, limiting accessibility of these therapies. In vivo generation of CAR T cells could overcome these barriers, but current methods rely either on transient expression with limited durability, or on random integration of DNA payloads that lack specificity. Here we demonstrate that stable and cell-specific transgene expression can be achieved through in vivo site-specific integration of large DNA payloads. We developed a two-vector system to deliver CRISPR–Cas9 ribonucleoproteins and a DNA donor template, using enveloped delivery vehicles and adeno-associated viruses, respectively. We optimized both vectors for T cell-specific delivery and gene-targeting efficiency. By integrating a CAR transgene into a T cell-specific locus, we generate therapeutic levels of CAR T cells in vivo in humanized mouse models of B cell aplasia, and haematological and solid malignancies. These findings offer a pathway to more efficient, precise and widely accessible T cell therapies.

## Abstract (web summary)

Stable and cell-specific transgene expression can be achieved through in vivo site-specific integration of large DNA payloads using a two-vector system of enveloped delivery vehicles and adeno-associated viruses.

## Main

CAR T cells are a promising treatment for haematological malignancies; to date, there are seven CAR T cell therapies that have been approved by the US Food and Drug Administration. Standard-of-care CAR T cell therapy requires patient-specific manufacturing, limited by variable product quality, long production times and high cost. CARs are usually delivered using retroviral vectors, producing heterogeneous expression from random integration^2,3^. Using CRISPR–Cas9 and adeno-associated virus (AAV)-mediated homology-directed repair (HDR), we targeted CAR integration into the endogenous human TCR alpha locus (*TRAC*). *TRAC*-CAR T cells display dynamic CAR expression that delays exhaustion and improves tumour control in xenograft and immunocompetent models^1,4^. This work has been critical for the development of allogeneic CAR T cell therapy, as it disrupts the TCR after transgene insertion—a necessary step to limit graft-versus-host disease (GvHD)^5^. Clinical trials using allogeneic *TRAC*-CAR T cells generated from healthy donors or induced pluripotent stem cells have achieved complete responses in patients with haematological malignancies when associated with deep lymphodepleting preconditioning^6^. Allogeneic approaches could address manufacturing limitations by creating off-the-shelf products from healthy donors. However, allogeneic CAR T cells are eventually rejected and frequent relapses have been observed^7^.

Direct in vivo CAR T cell generation may circumvent hurdles associated with leukapheresis and manufacturing. It might also promote the formation of a less differentiated CAR T cell pool^8^, a feature associated with improved antitumour activity^9–11^. Thus far, efforts to generate CAR T cells in vivo have used randomly integrating viral vectors for constitutive CAR expression or lipid nanoparticles (LNPs) that result in transient CAR expression^12–16^. Both modalities have been recently validated in non-human primates^17,18^ and, more recently, evaluated in a phase I trial^19^. These methods face challenges, including efficient gene delivery to therapeutic doses and risks of off-target transduction. Both delivery and CAR expression should be T cell specific, as off-target engineering of haematopoietic stem cells (HSCs) could lead to transformational mutagenesis^20^, and CAR expression in tumour cells could prevent cell surface expression of the CAR target and cause antigen-negative relapse^21^. LNP delivery of *CAR* mRNA leads to transient CAR expression, which prevents the risk of insertional mutagenesis or stable tumour cell expression, but required dosing remains unclear. Lentiviral vector envelopes can be engineered to provide improved specificity towards T cells^12,16,22–24^, but any transduced non-T cells would also express the CAR unless a lineage-specific promoter is used and, while rare, are at potential risk of insertional mutagenesis^25,26^. We hypothesized that integrating a promoterless *CAR* transgene at the *TRAC* locus in vivo would achieve T cell-specific and physiological CAR expression, while bypassing ex vivo cell manufacturing. To date, site-specific integration of large DNA payloads in human T cells in vivo remains elusive.

Here we develop a method combining AAVs with enveloped delivery vehicles (EDVs) to perform site-specific transgene integration in primary human T cells in vivo. By optimizing both the AAV and EDV tools for improved cell-specific delivery and resistance to human neutralizing antibodies, we were able to generate *TRAC*-CAR T cells in vivo at a therapeutic level and control tumour growth in several humanized mouse models.

## Targeted transgene integration in vivo

**Fig. 1:**
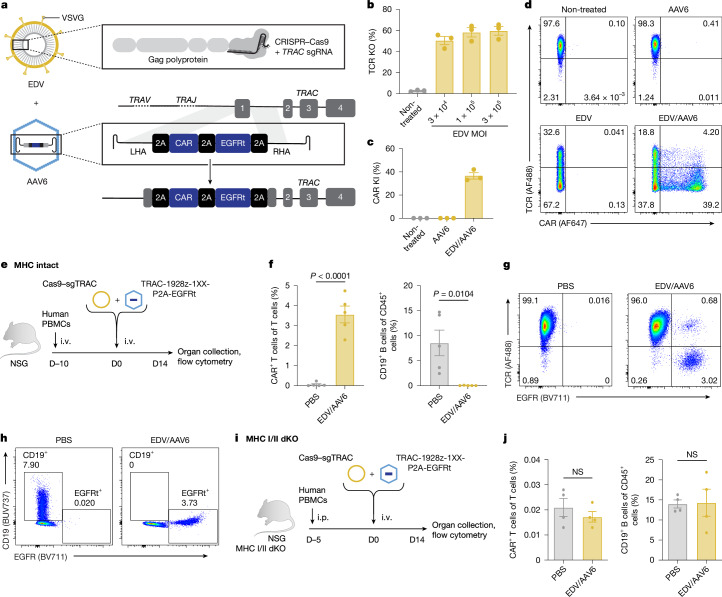
Co-delivery of Cas9-EDV and HDRT-AAV generates *TRAC*-CAR T cells in vitro and in vivo. **a**, A VSVG-WT-pseudotyped EDV delivering Cas9–RNP and an AAV6 delivering HDRT to knock in (KI) a CAR transgene at the *TRAC* locus. LHA, left homology arm; RHA, right homology arm. **b**, Activated human T cells transduced with Cas9–sgTRAC EDV at three different MOIs (based on sgRNA quantification). TCR-KO flow analysis at 72 h after transduction is shown. Data are mean ± s.e.m. *n* = 3 donors. **c**,**d**, Activated human T cells were treated with EDVs (Cas9–sgTRAC), AAV6 (*TRAC* CAR HDRT) or EDV + AAV6. CAR/TCR expression was assessed by flow cytometry at 72 h. Representative flow plots (**d**) and quantification (**c**) are shown. For **c**, data are mean ± s.e.m. *n* = 3 donors. **e**–**h**, MHC-intact NSG mice engrafted with human PBMCs (1 × 10^7^ cells) received PBS or EDV (2.5 × 10^11^ sgRNAs per mouse, Cas9–sgTRAC) and AAV6 (1 × 10^12^ vg per mouse, 1928z-1XX CAR-P2A-EGFRt *TRAC* HDRT). Spleens were collected on day 14 (D14) for flow cytometry. **e**, Experiment schematic. **f**, The percentage of CAR in T cells and CD19 in human CD45^+^ cells. Data are mean ± s.e.m. *n* = 5 mice per group **g**, Representative TCR/EGFR flow plot in splenic T cells from **f**. **h**, Representative CD19/EGFR expression in splenic CD45^+^ cells from **f**. **i**,**j**, MHC-I/II double-KO (dKO) NSG mice engrafted with human PBMCs (1 × 10^7^ cells) received PBS or EDV (2.5 × 10^11^ sgRNAs per mouse, Cas9–sgTRAC) and AAV6 (1 × 10^12^ vg per mouse, 1928z-1XX CAR-P2A-EGFRt *TRAC* HDRT). Spleens were collected on day 14 for flow cytometry. **i**, Experiment schematic. i.p., intraperitoneal. **j**, The percentage of CAR in T cells and CD19 among human CD45^+^ cells (**e**). Data are mean ± s.e.m. *n* = 4 mice per group. NS, not significant. Images in **e** and **i** were adapted from Servier Medical Art (https://smart.servier.com/), under a CC BY 4.0 licence. Source data

Site-specific integration in vivo requires delivery of a targeted endonuclease and HDR templates (HDRTs). Although EDVs have previously been shown to leverage antibody–antigen interactions to deliver Cas9–ribonucleoprotein (RNP) transiently and selectively to cells of interest in vivo^27^, nuclear delivery of large HDRTs in vivo remains a challenge. We used AAV6, which has been reported to transduce human T cells, natural killer (NK) cells and HSCs ex vivo^28–30^, to deliver a HDRT encoding a promoterless *CAR* cDNA flanked by self-cleaving 2A peptides and a truncated epidermal growth factor receptor (EGFRt)^1,28^. This design drives CAR expression from the endogenous *TRAC* promoter, which is T cell specific, after integration. To generate *TRAC*-CAR T cells, we combined a *TRAC*-targeted Cas9-EDV pseudotyped with the glycoprotein G from vesicular stomatitis virus (VSVG)^31^ and *TRAC*-CD19 CAR HDRT packaged in AAV6 (Fig. 1a).

**Extended Data Fig. 1:**
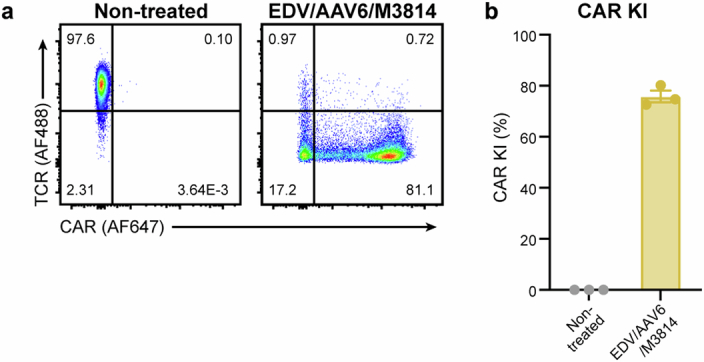
Related to main Fig. 1. **a**, Related to Fig. 1b–d. T cells were treated with a combination of EDV, AAV and 1 uM M3814 to improve. Representative flow plot showing TCR and CAR expression. **b**, CAR expression was determined by flow cytometry 72 h post transduction. Results are the mean ± SEM from three donors (n = 3). Source data

To assess Cas9–single guide RNA (sgRNA) delivery, we treated activated human primary T cells in vitro with the VSVG-EDV across a range of multiplicities of infection (MOI) and quantified TCR knockout (KO) using flow cytometry. EDV alone achieved 50% TCR KO even at the lowest MOI (3 × 10^4^ sgRNAs per cell), confirming efficacy previously reported^27^ (Fig. 1b). We next combined *TRAC*-targeted Cas9 EDVs with an AAV6 delivering a *TRAC*-CAR HDRT in primary T cells in vitro (3 × 10^5^ sgRNAs per cell EDV, 5 × 10^5^ viral genomes (vg) per cell AAV). EDV produced TCR KO in 67% of cells, while AAV6 alone had no effect as expected; however, AAV6/EDV treatment led to the emergence of a CAR^+^TCR^−^ population (39.2% of cells), indicating successful generation of *TRAC*-CAR T cells by this method in vitro (Fig. 1c,d). The DNA-PK inhibitor M3814 has been shown to improve the efficiency of HDR^32^, which was confirmed in our system, reaching up to around 80% knock-in (Extended Data Fig. 1a,b).

We next examined the efficacy of EDV/AAV treatment for *TRAC*-CAR T cell generation in vivo in a humanized mouse model. NSG mice were engrafted with human peripheral blood mononuclear cells (PBMCs), followed by an intravenous (i.v.) injection of *TRAC*-targeted Cas9 EDVs plus AAV6 *TRAC*-CAR HDRT carrying a CD19-28z-1XX CAR^33^ transgene followed by EGFRt (Fig. 1e). The 1XX architecture, in which the second and third immunoreceptor tyrosine-based activation motifs of the CD3 ζ chain are mutated, has been previously reported to improve CAR T cell accumulation and functional persistence in multiple tumour mouse models^33^. The mice were euthanized 14 days after treatment and the spleens were collected for flow cytometry analysis. Notably, around 3% of all splenic T cells in the treated mice were *TRAC*-CAR T cells (Fig. 1f,g), and the presence of CD19-28z-1XX CAR T cells was associated with B cell aplasia (Fig. 1f,h), demonstrating the functionality of in vivo generated CD19-targeting *TRAC*-CAR T cells. These experiments used MHC-I/II-intact NSG mice, in which human PBMCs induce xeno-GvHD. As HDR is dependent on cell cycling and EDV/AAV delivery is increased in activated T cells^34^, we speculated that the T cell activation provided by the xeno-GvHD might artificially inflate knock-in efficacy. To test this hypothesis, we used MHC-I/II double-KO NSG mice (Fig. 1i) in which xeno-GVHD is prevented, and we observed no detectable CAR T cells or splenic B cell aplasia (Fig. 1j). Thus, xeno-GvHD in MHC-intact NSG mice was required for VSVG-EDV and AAV6-mediated knock-in.

## Engineered vectors increase specificity and efficacy

We hypothesized that AAV6 and VSVG-EDV are limited by three major barriers: (1) the susceptibility of AAVs to neutralizing antibodies in the serum; (2) the lack of selectivity for T lymphocytes; and (3) the limited pool of cycling T cells.

**Fig. 2:**
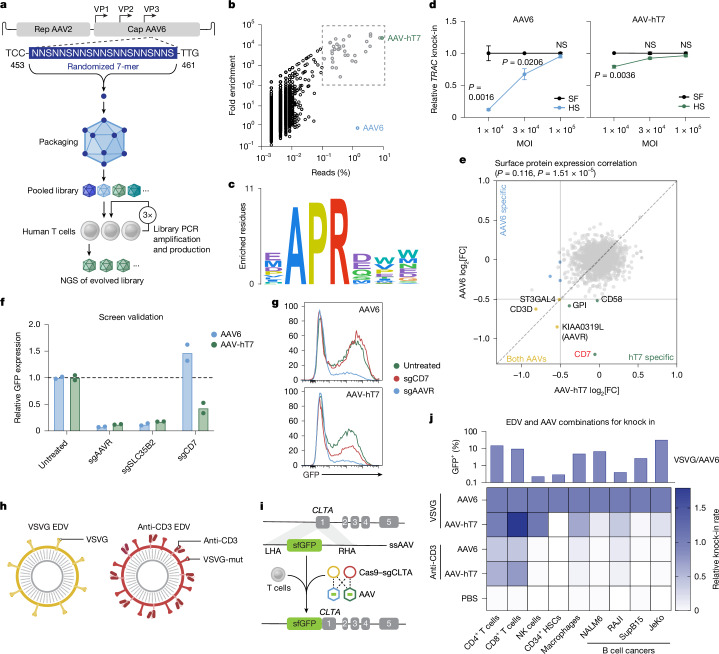
T cell-evolved AAV capsid and CD3-targeted EDVs improve delivery specificity. **a**, Schematic of AAV6 capsid library evolution through three selection cycles on activated human T cells cultured in human serum; parental and evolved libraries were analysed by NGS. **b**, NGS analysis of parental versus evolved libraries. Each variant is plotted by the fold enrichment in the evolved library versus parental (*y* axis) and the percentage of total evolved reads (*x* axis). Variants with >100-fold enrichment and >0.1% reads are highlighted. **c**, Sequence logo of the conserved 7-mer motif among top variants from **b**. **d**, Activated human T cells were electroporated with *TRAC*-targeting RNPs and treated with AAV6 or AAV-hT7 delivering *TRAC* CAR HDRT, in serum-free or human-serum-supplemented medium. CAR expression was measured using flow cytometry. Values are normalized to serum-free conditions within each MOI. Data are mean ± s.e.m. *n* = 3 donors. Statistical analysis was performed using multiple two-tailed unpaired* t*-tests with Holm–Šidák correction. **e**, Genome-wide KO screen for AAV-hT7 transduction, displaying cell surface genes. Correlation was assessed using the Spearman test with Benjamini–Hochberg-adjusted *P* values. FC, fold change. **f**,**g**, Activated T cells were electroporated with RNPs targeting *KIAA0319L*, *SLC35B2* or *CD7*, reactivated for 48 h, then transduced with AAV6 or AAV-hT7 encoding sc-CAG-GFP. The GFP mean fluorescence intensity (MFI) was measured using flow cytometry 48 h later and normalized to the untreated controls. **f**, Relative GFP MFI data are mean ± s.e.m.; *n* = 2 donors. **g**, Representative flow cytometry plots. **h**, Schematic of EDVs pseudotyped with WT VSVG (yellow) or mutated VSVG plus anti-CD3 scFv (anti-CD3, red). **i**,**j**, Cells were treated with VSVG-WT or anti-CD3 EDVs (Cas9–sgCLTA) and AAV6 or AAV-hT7 (*CLTA* exon 1 sfGFP HDRT) at 3 × 10^5^ sgRNA per cell and 5 × 10^5^ vg per cell. GFP indicates correct integration and was assessed using flow cytometry ≥72 h after treatment; integration was confirmed by dPCR.** i**, Schematic. For **j**, *n* = 3 (primary T cells), *n *= 2 (NK cells), *n* = 3 (CD34^+^ HSCs), *n* = 3 (macrophages) and *n* = 1 (four B cell lines). The heat map shows the relative knock-in normalized to VSVG-WT + AAV6 for which all cell types were positive for GFP signal (plotted above). Source data

**Extended Data Fig. 2:**
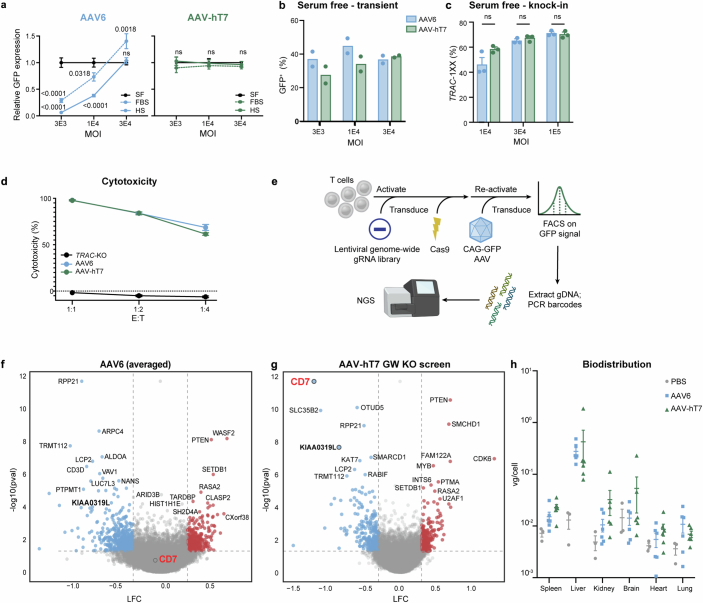
Related to main Fig. 2. **a**, Activated T cells were treated with either AAV6 or AAV-hT7 carrying a GFP transcript for transient expression. The cells were cultured in either serum free media (SF) or media supplemented with 10% fetal bovine serum (FBS) or human serum (HS) and GFP expression was measured by flow cytometry. Plotted values are GFP^+^ cells relative to the SF value in each MOI condition in order to highlight changes in transduction efficiency when serum is present. Results are the mean ± SEM from three donors (n = 3). Significance was assessed using two-way ANOVA and Dunnett’s multiple comparisons test. **b**, Activated T cells were treated with either AAV6 or AAV-hT7 carrying a GFP transcript for transient expression at three different MOI. The cells were cultured in either serum free media. These values are used to calculate the relative GFP expression in (Fig. 2d). Results are the mean from two donors (n = 2). **c**, Activated T cells from three donors were electroporated with an RNP targeting the *TRAC* locus and treated with either AAV6 or AAV-hT7 carrying an HDR template to knock in a CAR at *TRAC*. The cells were cultured in either serum free media. These values are used to calculate the relative GFP expression in (Fig. 2e). Results are the mean ± SEM from three donors (n = 3). Significance was assessed using multiple two-tailed unpaired *t*-tests and the Holm-Šidák multiple comparisons test. **d**, *TRAC*-CAR-T cells expressing a 1928z-1XX were generated with a combination of EDV and AAV and functionally validated. *TRAC*-CAR-T cells were co-culture NALM6 cells at three effector cells to tumour cell ratios (E:T) for 24 h. Cytotoxicity was calculated by a luminescence read-out. Results are the mean ± SEM from four technical replicates (n = 4). **e**, Schematic of a genome-wide knockout screen to identify genes associated with AAV-hT7 uptake and processing in primary T cells. T cells were isolated from PBMCs, activated with CD3/CD28 beads, and transduced with the lentiviral sgRNA library, followed by Cas9 electroporation (SLICE approach). 3 days later, T cells were re-activated for 48 h and transduced with AAV6 or AAV-hT7 expressing GFP. At 48 h after AAV-hT7 transduction cells were sorted into four bins based on GFP expression. Genomic DNA was extracted from cells in each bin, and amplicon libraries were prepared and sequenced to determine sgRNA enrichment. **f**,**g**, Volcano plot displaying genome-wide KO screen results for AAV6 (**f)** and AAV-hT7 (**g**), p-values determined using MAGeCK RRA one-sided tests and methods. **h**, PBMC-humanized were injected with either AAV6 (n = 6) or AAV-hT7 (n = 6) carrying a ss-CAG-GFP cargo, or PBS (n = 3). DNA was isolated from different organs harvested one week after injection. Viral genomes were quantified through qPCR and normalized by the DNA input. Results are the mean ± SEM. Source data

To address the sensitivity of AAV to neutralizing antibodies, we evolved an AAV6 capsid library using a strategy that we previously used to identify a mouse T cell-specific AAV^4^ (Fig. 2a). Human T cells were co-cultured with the capsid library at a low MOI in the presence of human serum, which is known to limit AAV6 transduction due to neutralizing antibody^35,36^. T cells were washed, and intracellular viral DNA was purified and cloned into the wild-type (WT) AAV plasmid backbone to generate a capsid library for a subsequent round of evolution. After three infection cycles, the parental and evolved libraries were analysed using next-generation sequencing (NGS), which identified dominant variants, including one with the amino acid motif HAPRVEE, displaying around 20,000-fold enrichment from the parental library (Fig. 2b). A sequence logo was generated based on the amino acid sequences of the variants that represented more than 0.1% of the reads in the evolved library and displayed more than a 100-fold enrichment from the parental library (Fig. 2b,c), revealing a conserved APR signature at amino acids 454 to 456 (Fig. 2c). We next assessed the evolved AAV for T cell transduction. We used AAV6 and the evolved AAV to deliver a self-complementary GFP expression cassette to primary human T cells and confirmed that AAV6 delivery was impaired at lower MOI in serum conditions. By contrast, the evolved AAV achieved delivery rates that were indistinguishable from serum-free conditions even at a low MOI, indicating resistance to pre-existing neutralizing antibodies (Extended Data Fig. 2a). Similarly, for *TRAC-*CAR HDRT delivery, AAV6 was sensitive to the presence of human serum in an MOI-dependent manner, whereas the evolved AAV achieved efficient *TRAC*-CAR knock-in at low MOI even in the presence of human serum (Fig. 2d and Extended Data Fig. 2a,b). *TRAC*-CAR T cells generated with either AAV6 or the evolved AAV were compared in a cytotoxicity assay against NALM6 cells, and we observed no differences (Extended Data Fig. 2c).

To identify host factors governing transduction of the newly evolved AAV variant, we performed a genome-wide CRISPR KO screen using the SLICE platform^37^ (Extended Data Fig. 2d). In brief, a pooled population of perturbed T cells was transduced with a GFP-expressing AAV packaged either as AAV6 or the T cell evolved AAV, then sorted into high-GFP and low-GFP bins for NGS (Extended Data Fig. 2e). Enrichment analysis of sgRNAs in the low-GFP fraction revealed genes required for AAV transduction (Fig. 2e and Extended Data Fig. 2f,g). The screens identified shared and vector-specific dependencies. As expected, *KIAA0319L* (also known as *AAVR*)—a well-established mediator of AAV entry and trafficking^38^—was depleted in both conditions. Similarly, *ST3GAL4* and other glycan-modifying genes were essential for efficient transduction of both AAV6 and the evolved variant^39^. As the screens used single-stranded AAVs, GFP expression also depended on second-strand synthesis and the transcriptional–translational machinery, as evidenced by depletion of sgRNAs encoding *CD3D* (TCR complex), *LCP2* (also known as SLP-76) and *RPP21*, genes that are essential for T cell activation and transcription. Importantly, the screen highlighted *CD7*, a transmembrane receptor expressed on T and NK cells, as the top determinant of transduction by the T cell evolved AAV (Fig. 2e and Extended Data Fig. 2f,g). To validate this dependency, we knocked out *CD7* as well as control genes known to influence AAV biology (*KIAA0319L* and *SLC35B2*, involved in glycan biosynthesis). While *KIAA0319L* and *SLC35B2* were required for both vectors, *CD7* loss markedly reduced transduction by the evolved variant but not by AAV6 (Fig. 2f,g). Hereafter, we refer to this variant, evolved on human T cells and targets CD7, as AAV-hT7. Finally, biodistribution analysis in our humanized mouse model revealed that AAV genomes from both AAV6 and AAV-hT7 could be detected in multiple tissues, including the liver and spleen, with no significant differences in tissue distribution (Extended Data Fig. 2h). However, note that histopathological analysis of these mice confirmed substantial infiltration of human T cells in the liver and other tissues, consistent with the expected distribution of human immune cells in this model.

A second barrier is a lack of T cell specificity at the delivery level. To further improve the selectivity of our EDV/AAV delivery system, we restricted the EDV specificity by incorporating a mutated VSVG with ablated affinity for the LDLR family of receptors^40^ and added an anti-CD3 single-chain variable fragment (scFv) that confers both targeting and activation^27^ (Fig. 2h). To confirm the specificity of our delivery method, we designed an assay in which our dual EDV/AAV treatment leads to the specific integration of a promoterless GFP in the first exon of the broadly expressed clathrin A (*CLTA*) gene through HDR^41^. As *CLTA* is broadly expressed by all cell types, GFP expression indicates integration due to uptake of both the AAV and EDV. We used combinations of the original and evolved AAV to deliver a template for GFP knock-in at *CLTA* with either VSVG or anti-CD3-pseudotyped EDVs to deliver *CLTA*-targeting Cas9–RNP (Fig. 2i). Primary human T cells, NK cells, macrophages, HSCs and a panel of human B cell cancer cell lines (NALM6, SupB15, JeKo1, Raji) were treated at a fixed MOI (3 × 10^5^ sgRNAs per cell EDV, 5 × 10^5^ vg per cell AAV) of EDV/AAV combinations for *GFP-CLTA* knock-in, and expression was assessed using flow cytometry (Fig. 2i,j).

**Extended Data Fig. 3:**
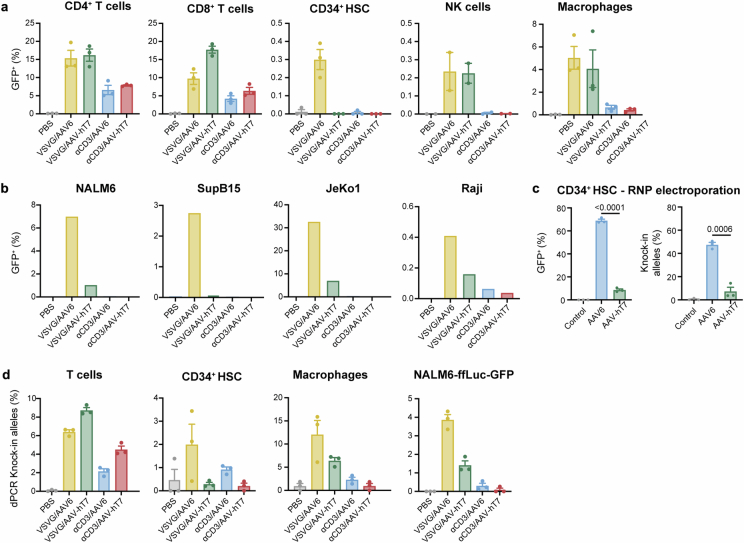
Related to main Fig. 2. **a**, Cells were treated with VSVG-WT or αCD3-EDVs carrying Cas9 and a sgRNA targeting *CLTA*, and AAV6 or AAV-hT7 carrying a HDR template to knock-in a reporter sfGFP transcript in exon 1 of *CLTA*. GFP expression is only observed if correctly integrated in *CLTA*. Cells were treated with 3 × 10^5^ sgRNA/cell EDV, and with 5 × 10^5^ vg/cell AAV. GFP expression was analysed by flow cytometry at least 72 h after treatment and genomic integration was confirmed by dPCR. Primary human T cells (n = 3), NK cells (n = 2), CD34^+^ HSC (n = 3), Macrophages (n = 3). Results are the mean ± SEM from two or three donors (n = 2 or 3). **b**, Four cell lines from B cell malignancies NALM6, Raji, SupB15 JeKo were treated according to **d**. GFP expression was analysed by flow cytometry at least 72 h after treatment. **c**, Human CD34^+^ hematopoietic stem cells were electroporated with Cas9/sg*CLTA* and treated with AAV6 or AAV-hT7 carrying a HDR template to knock-in a reporter sfGFP transcript in exon 1 of *CLTA*. Cells were treated 5 × 10^4^ vg/cell AAV. GFP expression was analysed by flow cytometry at least 72 h after treatment (left panel) and genomic integration was confirmed by dPCR (right panel). Results are the mean ± SEM from three donors (n = 3). Significance was assessed using a two-tailed unpaired *t*-test. **d**, dPCR analysis on genomic DNA extracted from samples in (**d**) to confirm genomic integration at *CLTA*. Including knock-in analysis of treated NALM6-ffLuc-GFP cells treated the same way as **d** (flow cytometry analysis not available due to ubiquitous GFP expression). Data presented as percent of edited alleles. Results are the mean ± SEM from three donors (n = 3). Source data

VSVG/AAV6 showed broad tropism, with significant GFP^+^ populations across all of the tested cell lineages (Fig. 2j and Extended Data Fig. 3a,b). AAV-hT7 abolished targeting of HSCs, and the combination of AAV-hT7/anti-CD3-EDV conferred selectivity for CD4^+^ and CD8^+^ T cells, despite an overall lowered efficiency when using anti-CD3-EDV (Extended Data Fig. 3a). As HSCs did not express the receptors to take up our EDV designs, we compared the efficacy of HDRT delivery using AAV6 and AAV-hT7 after RNP electroporation and observed a significant decrease in *GFP-CLTA* knock-in when using AAV-hT7 (Extended Data Fig. 3c), confirming that AAV-hT7 is detargeted from human HSCs. Although VSVG/AAV6 knock-in efficiency was low in NK cells, it remained unchanged with the addition of AAV-hT7 (Fig. 2j), which is consistent with NK cells expressing CD7. NK cell knock-in was abolished when using the anti-CD3-EDV. Importantly, CAR expression in tumour cells after in vivo delivery of gene editing particles could prevent cell surface expression of the CAR target and cause antigen-negative relapse, as it has been reported in autologous settings^21^. Thus, it was encouraging that AAV-hT7 reduced the knock-in efficiency in all of the B cell cancer cell lines tested, and the combination of anti-CD3-EDV/AAV-hT7 completely abolished cargo integration in cancer cells (Fig. 2j and Extended Data Fig. 3b). The efficiencies of integration measured by flow cytometry were further confirmed by digital PCR (dPCR) using genomic DNA (Extended Data Fig. 3d).

**Extended Data Fig. 4:**
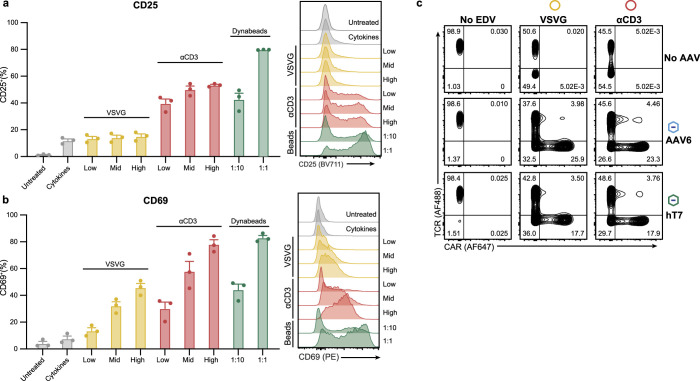
Related to main Fig. 2. **a**, Naive human T cells were treated with either VSVG-WT or αCD3-EDVs carrying Cas9/sg*TRAC* at three MOI (low 3 × 10^4^ sgRNA/cell, mid 1 × 10^5^ sgRNA/cell or high 3 × 10^5^ sgRNA/cell). Untreated naive T cells and T cells treated with only cytokines (IL7/IL15) were used as control samples. T cells treated with dynabeads at a 1:1 or 1:10 bead to cell ratio were included as positive controls. CD25 expression was determined by flow cytometry 48 h post transduction. Results are the mean ± SEM from three donors (n = 3). Right panel, representative donor displaying CD25 expression as summarized in (**a**). **b**, CD69 expression determined by flow cytometry 48 h post transduction in samples from (**a**). Results are the mean ± SEM from three donors (n = 3). Right panel, representative donor displaying CD69 expression as summarized in (**b**). **c**, Activated human T cells were treated with either VSVG-WT or αCD3-EDVs carrying Cas9/sg*TRAC* and with AAV6 or AAV-hT7 carrying an HDR template to knock-in a CAR at *TRAC* (EDV at 3 × 105 sgRNA/cell and AAV at of 5 × 105 vg/cell). TCR and CAR expressions were determined by flow cytometry 72 h post transduction. Source data

Finally, to increase the pool of cycling T cells in vivo, we tested whether anti-CD3-EDV could induce activation of T cells. Naive T cells were transduced with either VSVG or anti-CD3-EDV at three MOIs, and were analysed for the expression of the activation markers CD25 and CD69. T cells treated with anti-CD3/anti-CD28 Dynabeads were used as a positive control for activation. We observed a robust increase in CD25 and CD69 expression after transduction with anti-CD3-EDV comparable to treatment with anti-CD3/anti-CD28 Dynabeads at a 1:10 bead to cell ratio. By contrast, VSVG-EDV induced mild levels of CD69 and no CD25 expression, indicating that the anti-CD3 scFv displayed at the surface of EDVs is sufficient to induce activation in naive T cells (Extended Data Fig. 4a,b).

Together, the evolved AAV capsid and anti-CD3-EDV address the three established challenges to in vivo delivery.

## Anti-CD3-EDV and AAV-hT7 improve in vivo knock in

We next assessed the AAV-hT7/anti-CD3-EDV combination for in vitro *TRAC*-CAR T cell generation. As expected, AAV alone had no effect, while EDV alone induced 50% TCR KO (Extended Data Fig. 4c). All combinations of AAV and EDV generated TRAC-CAR T cells in vitro.

**Fig. 3:**
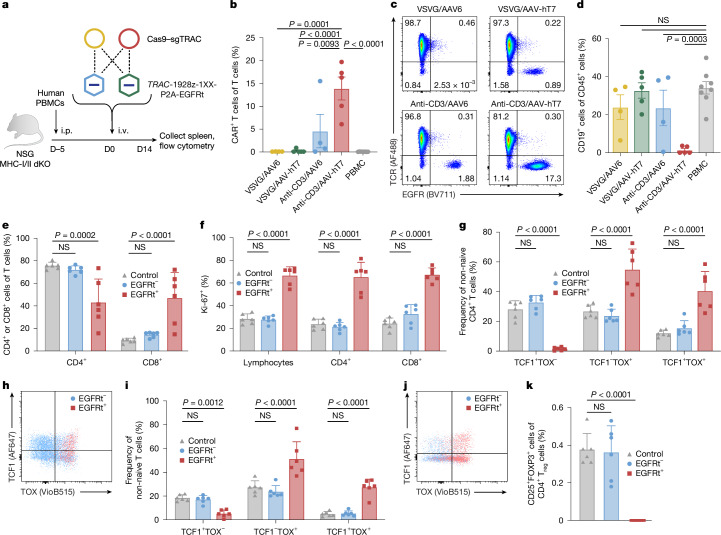
Improved in vivo generation of functional *TRAC*-CAR T cells. **a**, NSG MHC-I/II double-KO mice engrafted with human PBMCs (1 × 10^7^) received PBS or EDVs (VSVG-WT or anti-CD3; 2.5 × 10^11^ sgRNAs per mouse; Cas9–sgTRAC) plus AAVs (AAV6 or AAV-hT7; 1 × 10^12^ vg per mouse) delivering a *TRAC* HDRT encoding the 1928ζ-1XX-CAR-P2A-EGFRt. Spleens were collected on day 14 for flow cytometry analysis. **b**, CAR expression in T cells. Data are mean ± s.e.m. *n* = 8 (PBS), *n* = 4 (VSVG/AAV6), *n* = 5 (VSVG/AAV-hT7), *n* = 4 (anti-CD3/AAV) and *n* = 5 (anti-CD3/AAV-hT7). **c**, Representative CAR/TCR flow cytometry plot from **b**. **d**, CD19 expression in human CD45^+^ splenocytes. Data are mean ± s.e.m. The group sizes were as described in **b**. **e**–**k**, Spectral flow phenotyping of human CD45^+^ splenocytes. Analyses included all samples with sufficient cell numbers. PBS-treated mice were used as controls (*n* = 6). CAR-treated samples included one anti-CD3/AAV6 and five anti-CD3/AAV-hT7 mice (*n* = 6 total). Within each mouse, TRAC-CAR T cells (EGFRt^+^) were compared with unedited T cells (EGFRt^−^). Data are mean ± s.e.m. *n* = 6. **e**, CD4^+^ and CD8^+^ T cell frequencies. **f**, The Ki-67^+^ frequencies in total lymphocytes and CD4^+^ and CD8^+^ T cells. **g**,**h**, Representative flow cytometry plot (**f**) and quantification (**h**) of TOX and TCF1 expression in non-naive CD4^+^ T cells. **i**,**j**, Representative flow cytometry plot (**j**) and quantification (**i**) of TOX and TCF1 expression in non-naive CD8^+^ T cells.** k**, The frequency of regulatory T cells (FOXP3^+^CD25^+^) among CD4^+^ T cells. Statistical analysis was performed using two-way analysis of variance (ANOVA) with Tukey’s multiple-comparison test (**b**,**d**) and two-way ANOVA with Dunnett’s multiple-comparison test (**e**–**k**). Images in **a** were adapted from Servier Medical Art (https://smart.servier.com/), under a CC BY 4.0 licence. Source data

**Extended Data Fig. 5:**
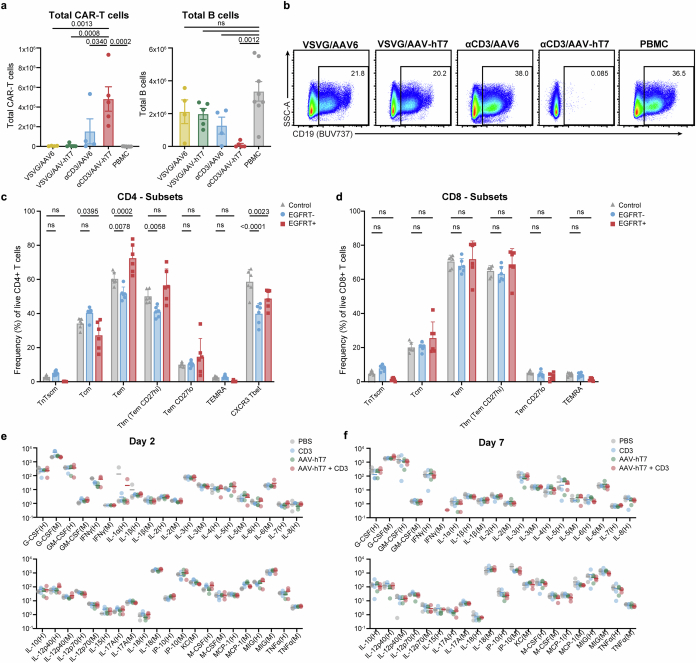
Related to main Fig. 3. **a**, From Fig. 3a. NSG-MHCI/II dKO mice transplanted with human PBMC (1 × 10^7^ cells) were injected with either PBS or EDV (VSVG-WT or αCD3, 2.5 × 10^11^ sgRNA per mouse) carrying Cas9/sg*TRAC* and AAV (AAV6 or AAV-hT7, 1 × 10^12^ vg per mouse) carrying an HDR template targeting 1928z-1XX and EGFRT to *TRAC*. Spleens were harvested and isolated for flow cytometry analysis 14 days after EDV/AAV injections. CAR and CD19 expression in T cells were determined by flow cytometry in spleens. Left panel, total CAR-T cells in each spleen. Right panel, total B cells in each spleen. Results are the mean ± SEM from four to eight mice per group (n = 4,5 or 8). **b**, Representative flow plot from (Fig. 3d) displaying CD19 expression in human CD45^+^ cells. **c**, Spectral flow analysis on CD4^+^ T cell subsets in control, *TRAC*-CAR-T cells (EGFRT^+^) and unedited T cells (EGFRT^−^), data related to (Fig. 3e-k). Results are the mean ± SEM from six mice per group (n = 6). **d**, Spectral flow analysis on CD8^+^ T cell subsets in control, *TRAC*-CAR-T cells (EGFRT^+^) and unedited T cells (EGFRT^−^), data related to (Fig. 3e–k). Results are the mean ± SEM from six mice per group (n = 6). **e**,**f**, Peripheral blood samples were collected from three mice (n = 3) injected with either PBS, EDV (CD3), AAV (AAV-hT7) or EDV/AAV (AAV-hT7 + CD3), on day 2 (e) or day 7 (f) after injection. A multi-marker panel was measured using Eve Technologies Humanized Mouse 41-Plex Discovery Assay Array (HUMU41). **a**, Significance was assessed using two-way ANOVA and Tukey’s multiple comparisons test. **c**,**d**,Significance was assessed using two-way ANOVA and Dunnett’s multiple comparisons test. Source data

**Extended Data Fig. 6:**
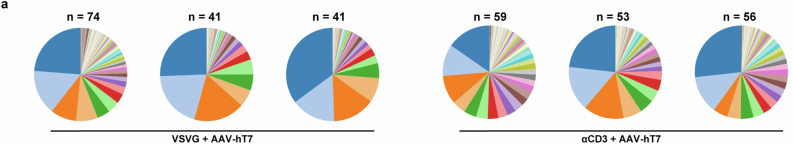
Related to main Fig. 3. **a**, Clonal analysis on T cells isolated from mice described in Fig. 3a (n = 3). Splenic CAR-T cells were isolated 14 days after EDV/AAV treatment and were analysed using SMART Human TCR a/b Profiling Kit from Takara Bio. The number of clones identified (with a cutoff at >0.001% of the total reads) is annotated for each sample (n). Each colour represents a unique clone and its proportion of reads in the pie chart and is sorted based on clone rank. Source data

We next tested whether in vitro improvements translated in the xeno-GvHD-free humanized mouse model. Four EDV/AAV combinations composed of VSVG/anti-CD3 pseudotyped EDVs combined with AAV6/AAV-hT7 were tested. Five days after injection of human PBMCs, we delivered AAVs carrying a CD19-28z-1XX-P2A-EGFRt *TRAC*-HDRT along with EDVs containing *TRAC* Cas9–RNP (Fig. 3a). Two weeks after vector injection, the mice were euthanized and the spleens were collected for flow cytometry analysis. We observed that anti-CD3-EDV significantly improved *TRAC*-CAR T cell generation when combined with AAV6, averaging around 5% of all splenic T cells. We observed significantly more CAR T cells in the optimized anti-CD3/AAV-hT7-treated mice compared with in all of the other groups, with CAR integrations in up to 19.7% of all splenic T cells (Fig. 3b,c). CAR T cell numbers were significantly higher after use of AAV-hT7 compared with AAV6, with a threefold increase in absolute CAR T cell numbers over AAV6 when either is combined with the anti-CD3-EDV (Extended Data Fig. 5a). Furthermore, only the mice treated with the anti-CD3/AAV-hT7 resulted in systematic complete B cell aplasia (Fig. 3d and Extended Data Fig. 5b). Analysis of serum cytokines at 1 and 7 days after vector (AAV or EDV) administration showed no evidence of systemic inflammation or cytokine release (Extended Data Fig. 5e,f). TCR clonality analysis of spleen samples revealed a more limited distribution compared with that of ex vivo manufactured autologous CAR T cells, but the data are consistent with previous in vivo engineering studies^27,42^ (Extended Data Fig. 6a). Together, these results demonstrate that anti-CD3-EDV is essential for in vivo *TRAC*-CAR T cell generation, and that the evolved AAV-hT7 vector confers markedly improved transduction efficiency and functional potency compared with AAV6, resulting in complete B cell depletion and robust CAR T cell expansion.

To further characterize in vivo generated *TRAC*-CAR T cells, we performed spectral flow analysis. We compared three T cell groups: T cells from untreated control mice, and CAR-positive and CAR-negative T cells (determined by EGFRt expression) from the same mice treated with EDV/AAV (five mice with anti-CD3/AAV-hT7 and one mouse with anti-CD3/AAV6). For the analysed markers, we observed no differences between untreated mice and CAR-negative T cells (Fig. 3e–k). This suggests that vector injection had a limited effect on T cell phenotypes. In the *TRAC*-CAR T cell population, we confirmed the ability to engineer both CD4^+^ and CD8^+^ T cells in vivo, with a balanced CD4-to-CD8 ratio (Fig. 3e). As the control mice and EGFRt-negative population have a higher CD4^+^ population, this result suggests that there is either a higher initial pool of engineered CD8^+^ T cells or a greater proliferation of this subset. Notably, while CD8^+^ subsets showed no difference, we observed a reduction in the central-memory-associated subsets with an increase in effector memory subsets among CD4^+^ CAR T cells (Extended Data Fig. 5c,d). Furthermore, we found that Ki-67 expression was significantly higher in *TRAC*-CAR T cells compared with in both control cells and unedited T cells, in both CD4^+^ and CD8^+^ subsets, suggesting the generation of a highly proliferative cell population (Fig. 3g). Finally, we assessed TCF1 and TOX expression within *TRAC*-CAR T cells and observed a substantial TOX-positive population, with 27.4% maintaining TCF1 expression (Fig. 3h–k). Together, this analysis suggests the generation of a highly proliferative CAR T cell population that maintains memory markers and displays a phenotype attributed to progenitor exhausted T cells, a feature probably associated with the 1928z-1XX architecture^33^. Finally, when targeting T cells in vivo, there is a risk of endowing regulatory T cells with the CAR transgene, which has been shown to affect the response rate in haematological malignancies^43^. Importantly, our data show a significantly reduced frequency of regulatory T cells in CAR^+^CD4^+^ cells compared with the control population (Fig. 3k).

Together, these results demonstrate successful *TRAC*-CAR T cells generation in vivo, representing, to our knowledge, the first targeted integration of a large DNA payload in primary human T cells in vivo.

## In vivo *TRAC*-CAR T cell control of B-ALL

**Fig. 4:**
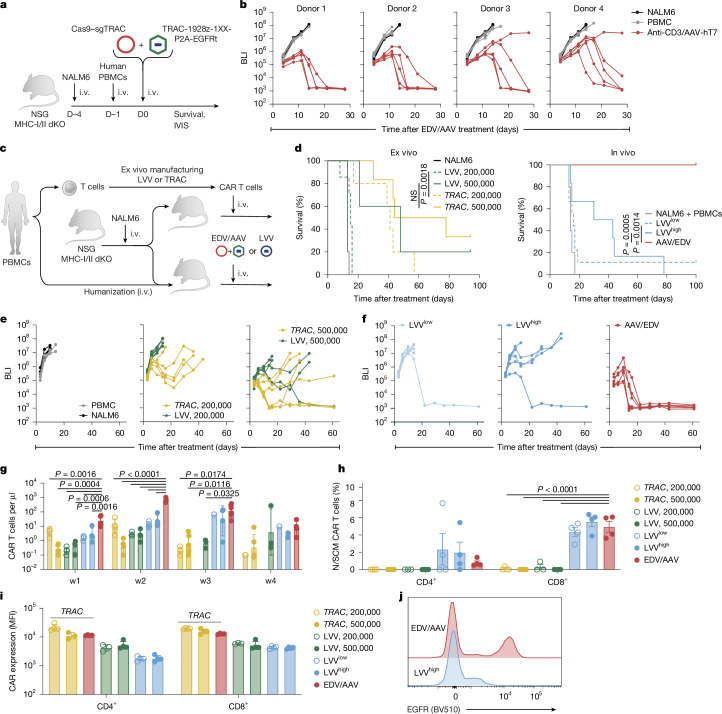
In vivo *TRAC*-targeted CAR T cells outperform in vivo lentiviral CAR T cells in a B-ALL model. **a**, Tumour challenge schematic. NSG MHC-I/II double-KO mice received i.v. NALM6-ffLuc-GFP cells (5 × 10^5^), followed 3 days later by human PBMCs (1 × 10^7^). Then, 1 day after PBMC transfer, mice received PBS or anti-CD3-EDV (5 × 10^11^ sgRNAs per mouse; Cas9–sgTRAC) plus AAV-hT7 (1 × 10^12^ vg per mouse; 1928ζ-1XX-CAR-P2A-EGFRt *TRAC* HDRT). The tumour burden was monitored using bioluminescence imaging (BLI). IVIS, in vivo imaging system. **b**, BLI measurements in mice injected with NALM6 alone (*n* = 5), NALM6 + PBMC (*n* = 5) or NALM6 + PBMC + EDV/AAV (*n* = 5). Experiments were repeated using four PBMC donors. BLI values represent the mean of dorsal and ventral signals (photons per s per cm^2^). **c**, Schematic comparing ex vivo and in vivo engineered CAR T cells. NALM6-challenged mice received either PBMCs (followed by EDV/AAV or LVVs expressing 1928ζ−1XX CAR) or ex vivo generated CAR T cells derived from the same donor PBMCs. The tumour burden was tracked using BLI. **d**, Kaplan–Meier survival analysis. Left, mice receiving NALM6 only (*n* = 5) or ex vivo *TRAC*-CAR T or lentiviral CAR T cells at 2 × 10^5^ or 5 × 10^5^ cells (*TRAC*, *n* = 5 and 6; LV, *n* = 7 and 5). Right, mice receiving NALM6 + PBMC with PBS (*n* = 5), EDV/AAV (*n *= 6), LVV^low^ (*n* = 9), or LVV^high^ (*n* = 6). Statistical analysis was performed using the log-rank (Mantel–Cox) test. **e**,**f**, BLI measurements from mice in **c** and **d** (ex vivo (**e**) and in vivo (**f**)). Values are the average of dorsal and ventral signals (photons per s per cm^2^). **g**–**j**, Flow cytometry analysis of peripheral blood collected from the mice described in **c** and **d**.**g**, Longitudinal peripheral blood CAR T cell expansion. Data are geometric mean ± geometric s.d. **h**, The frequency of naive/stem cell memory (N/SCM) (CD45RA^+^CD62L^+^) CAR T cells at week 2, separated by CD4^+^ and CD8^+^ subsets. Data are mean ± s.e.m. **i**,**j**, Quantification (**i**) and representative flow cytometry plots (**j**) of CAR expression (EGFRt MFI) at week 2 in CD4^+^ and CD8^+^ CAR T cells. Data are geometric mean ± geometric s.d. Statistical analysis was performed using one-way ANOVA with Dunnett’s multiple-comparison test (**g**–**i**). Images in **a** and **c** were adapted from Servier Medical Art (https://smart.servier.com/), under a CC BY 4.0 licence. Source data

**Extended Data Fig. 7:**
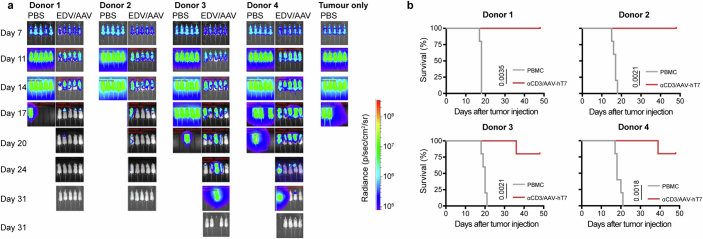
Related to main Fig. 4. **a**, Dorsal images from mice described in Fig. 4a. BLI is displayed as radiance (p/sec/cm^2^/sr). Days are indicated from time of PBS or EDV/AAV treatment. Day 27 is shown with and without mice that are saturating the signal. **b**, Kaplan-Meier survival analysis of mice described in Fig. 4a,b. Days are counted from NALM6 injections (5 × 10^5^ cells). Mice were transplanted with PBMCs (1 × 10^7^ cells) from four donors and treated with either PBS (n = 5) or EDV/AAV (n = 5). Significance was assessed using a log-rank (Mantel-Cox) test. Source data

**Extended Data Fig. 8:**
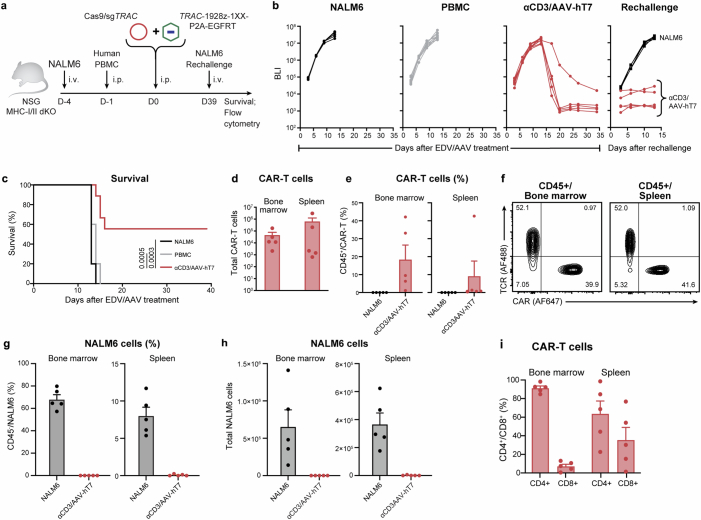
Related to main Fig. 4. **a**, Schematic of tumour challenge model. NSG-MHCI/II dKO mice were injected with 2.5 × 10^5^ NALM6-ffLuc-GFP cells intravenously, followed by an intraperitoneal injection of 2 × 10^7^ human PBMCs three days later. One day following the PBMC injections, the mice were injected with either PBS (PBMC only) or αCD3-EDV (2.5 × 10^11^ sgRNA per mouse) and AAV-hT7 (1 × 10^12^ vg per mouse) carrying an HDR template targeting 1928z-1XX CAR and EGFRT to *TRAC*. Tumour burden was tracked by bioluminescence imaging (BLI) measurements. At day 39 post EDV/AAV injection, the mice were rechallenged with 5 × 10^6^ NALM6-ffLuc-GFP cells intravenously and euthanized for organ harvest after 13 days. **b**, BLI measurements in mice injected with NALM6 (n = 5), NALM6 and PBMC (n = 10), or NALM6, PBMC and EDV/AAV (n = 9). Rechallenge with NALM6 was performed in five EDV/AAV treated mice that controlled tumour growth by day 39, and in five age-matched control mice. BLI values are the average of dorsal and ventral signals and presented as photons/s/cm^2^. **c**, Kaplan-Meier survival analysis until day 39 comparing mice injected with NALM6 only (n = 5), NALM6 and PBMC (n = 10), or NALM6, PBMC and EDV/AAV (n = 9). Significance was assessed using a log-rank (Mantel-Cox) test. **d**, Total number of CAR-T cells in bone marrow and spleen in NALM6 rechallenged mice from (**a**,**b**). CAR expression was determined by flow cytometry. Results are the mean ± SEM from five mice (n = 5). **e**, CAR-T cell percentages in human CD45^+^ cells from bone marrow and spleen in NALM6 rechallenged mice from (**a**,**b**). CD45 and CAR expressions were determined by flow cytometry. Results are the mean ± SEM from six mice (n = 5). **f**, Representative flow plots showing TCR and CAR expression in human CD45^+^ cells from bone marrow and spleen from (**d**). **g**, NALM6 (CD19^+^/GFP^+^/CD45^−^) percentages in bone marrow and spleen in NALM6 rechallenged mice from (**a**,**b**). CD45, GFP and CD19 expressions were determined by flow cytometry. Results are the mean ± SEM from six mice (n = 5). **h**, Total number of NALM6 cells (CD19^+^/GFP^+^/CD45^−^) bone marrow and spleen in NALM6 rechallenged mice from (**a**,**b**). CD45, GFP and CD19 expressions were determined by flow cytometry. Results are the mean ± SEM from six mice (n = 5). **i**, CD4 and CD8 expression in CAR-T cells from bone marrow and spleen in NALM6 rechallenged mice from (**a**,**b**) as determined by flow cytometry. Results are the mean ± SEM from five mice (n = 5). Source data

To assess the functional capacity of in vivo-generated CAR T cells to control tumours in a model of B-cell acute lymphoblastic leukaemia (B-ALL), we first challenged NSG-MHC-I/II double-KO mice with a NALM6 aggressive leukaemia cell line, followed by PBMCs 3 days later (Fig. 4a). EDV/AAV were injected 1 day after PBMC injection and, as we previously demonstrated that the anti-CD3/AAV-hT7 combination was the only one to achieve robust B cell aplasia, we proceeded with only this treatment. We performed these experiments with four PBMC donors to assess donor variability. After a single injection, 18 out of 20 mice across four donors achieved complete responses (Fig. 4b and Extended Data Fig. 7a,b). In an experiment with a fifth PBMC donor, mice that controlled the tumour were rechallenged with NALM6 (5 × 10^6^ cells) at day 39 after treatment and euthanized at day 52 for organ collection and flow cytometry analysis (Extended Data Fig. 8a). EDV/AAV-treated mice controlled the rechallenge, with no tumour increase over a 2-week period after rechallenge (Extended Data Fig. 8b,c).

Analysis of the rechallenged mice demonstrated high numbers of total CAR T cells in both the bone marrow and spleen and elimination of NALM6 cells in these organs (Extended Data Fig. 8d–h). Up to 40% of human CD45^+^ cells were found to be CAR^+^ after EDV/AAV delivery (Extended Data Fig. 8e). Furthermore, we observed both CD8^+^ and CD4^+^ CAR T cells in both bone marrow and spleen of the mice injected with EDV/AAV, with a more dominant CD4^+^ population in the bone marrow compared with in the spleen (Extended Data Fig. 8i).

To benchmark in vivo generated *TRAC*-CAR T cells against conventional CAR T cell therapies, we compared *TRAC*-integrated and lentivirally expressed CARs produced either ex vivo or in vivo. *TRAC-*CAR T cells and lentiviral CAR T cells were manufactured from the same PBMC donor, frozen and then infused into NALM6-bearing NSG-MHC-I/II double-KO mice at two doses (2 × 10^5^ or 5 × 10^5^ CAR T cells) after thawing (Fig. 4c). Parallel cohorts were humanized with the same donor PBMCs before in vivo engineering using either anti-CD3-pseudotyped lentiviral vectors (LVV) or EDV/AAV (Fig. 4c). LVVs were administered at two levels: one matched to the effective transduction units of EDV/AAV (LVV^low^) and one matched by viral particle number (LVV^high^, 3× higher than LVV^low^). For optimal comparisons, all T cells were engineered to express the same 1928z-1XX CAR.

Ex vivo manufactured *TRAC-*CAR T cells displayed improved tumour control compared with lentiviral CAR T cells at the low dose (2 × 10^5^ CAR T cells), but no significant difference was observed at a high dose (5 × 10^5^ CAR T cells) (Fig. 4d,e). For in vivo engineering, while the efficacy was limited in mice treated with the low doses of LVV, 4 out of 6 LVV-treated mice responded at a high dose, with only one showing complete remission (Fig. 4d,e). Complete tumour control occurred in 6 out of 6 mice with EDV/AAV treatment, while either lentiviral treatment showed only a partial or limited response (Fig. 4d–f).

Longitudinal blood profiling revealed marked differences in CAR T cell expansion dynamics (Fig. 4g). In vivo engineered *TRAC*-CAR T cells expanded rapidly (red bars), peaking at week 2, while in vivo LVV CAR T cells peaked at week 3 (blue bars). The expansion of in vivo *TRAC*-CAR T cells at week 1 was 8- or 20-fold higher compared with LVV^low^ or LVV^high^, respectively, and at week 2 it was 21- or 50-fold higher compared with LVV^low^ or LVV^high^, respectively (Fig. 4g). After tumour clearance, *TRAC-*CAR T cells contracted in the circulation, consistent with normal effector dynamics.

**Extended Data Fig. 9:**
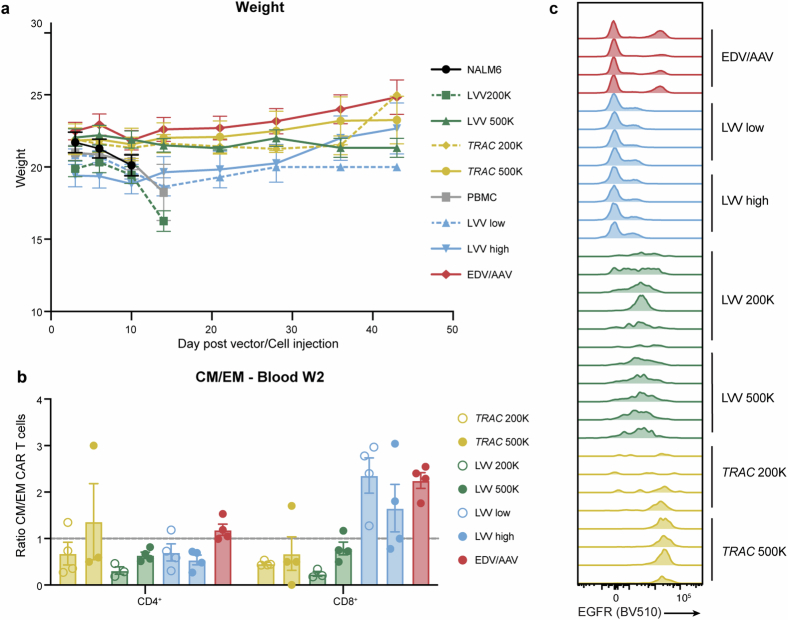
Related to main Fig. 4. **a**, Summary of total body weight measurements from mice in Fig. 4c. **b**, Ratio of CM (CD45RA−/CD62L+) and EM (CD45RA+/CD62L−) cells within CD4^+^ or CD8^+^ CAR-T cells in peripheral blood two weeks after injection in Fig. 4c. **c**, Representative flow plot showing EGFR expression in samples from Fig. 4i. Source data

In vivo engineered CAR T cells across all conditions displayed a higher frequency of the CD45RA^+^CD62L^+^ double-positive CD8^+^ subset, indicative of naive or stem cell memory phenotypes (Fig. 4h and Extended Data Fig. 9b). All *TRAC*-CAR T cell products displayed high and homogeneous CAR expression, in contrast to the lower and more variable expression observed with lentiviral CAR T cells (Fig. 4j and Extended Data Fig. 9c), probably reflecting the uniform transcriptional control conferred by site-specific integration at the *TRAC* locus.

Overall, these results demonstrate that our engineered AAV and EDV can be used in vivo for site-specific integration of a CAR to generate *TRAC*-CAR T cells that are highly effective in the treatment of an aggressive B cell leukaemia model.

## In vivo TRAC-CAR T cells in myeloma and sarcoma

**Fig. 5:**
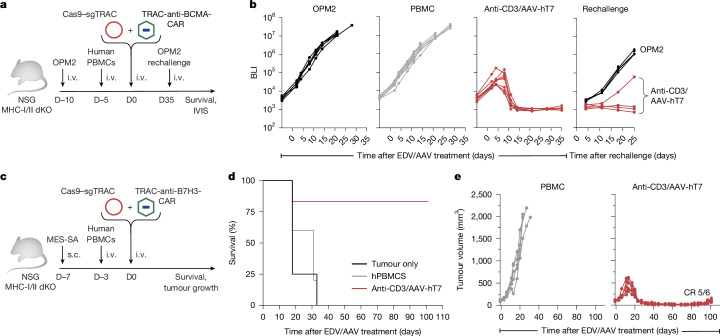
In vivo generated *TRAC*-CAR T cells confer antitumour activity in haematological and solid tumour models. **a**, Tumour challenge schematic for multiple myeloma. NSG MHC-I/II double-KO mice received i.v. OPM2 cells (1 × 10^6^), followed 5 days later by human PBMCs (1 × 10^7^). Then, 5 days after PBMC transfer, mice received PBS or anti-CD3-EDV (5 × 10^11^ sgRNAs per mouse; Cas9–sgTRAC) plus AAV-hT7 (1 × 10^12^ vg per mouse), delivering a TRAC HDRT encoding a BCMA-1XX-CAR-P2A-EGFRt. The tumour burden was monitored using BLI. At day 35 after EDV/AAV treatment, mice were rechallenged i.v. with OPM2 cells (5 × 10^6^). **b**, BLI measurements in mice injected with OPM2 alone (*n* = 5), OPM2 + PBMC (*n* = 8) or OPM2 + PBMC + EDV/AAV (*n* = 8). Rechallenge was performed in four EDV/AAV-treated mice that controlled tumour growth by day 35 and in five age-matched control mice. BLI values represent the mean of dorsal and ventral signals (photons per s per cm^2^). **c**, Tumour challenge schematic for solid tumours. NSG MHC-I/II double-KO mice received subcutaneous (s.c.) MES-SA cells (4 × 10^6^), followed 4 days later by human PBMCs (1 × 10^7^). Then, 3 days after PBMC transfer, mice received PBS or anti-CD3-EDV (5 × 10^11^ sgRNAs per mouse) plus AAV-hT7 (1 × 10^12^ vg per mouse), delivering a *TRAC* HDRT encoding an anti-B7H3-CD28ζ-1XX-CAR-P2A-EGFRt. The tumour burden was assessed by calliper measurements.** d**, Kaplan–Meier survival analysis of mice bearing MES-SA tumours; MES-SA only (*n* = 4), MES-SA + PBMC (*n* = 5) or MES-SA + PBMC + EDV/AAV (anti-B7H3–CD28ζ−1XX TRAC CAR; *n* = 6). **e**, Tumour growth measurements from the mice in **d**. CR, complete response. Images in **a** and **c** were adapted from Servier Medical Art (https://smart.servier.com/), under a CC BY 4.0 licence. Source data

Encouraged by these results, we next evaluated the potential of in vivo generated *TRAC-*CAR T cells beyond CD19-directed settings, in which B cell antigen drives natural CAR T expansion. We first engineered a *TRAC*-BCMA-CAR donor (HDRT) and packaged it into AAV-hT7. NSG-MHC-I/II double-KO mice were engrafted with the multiple myeloma cell line OPM2, followed by PBMC injection and EDV/AAV treatment (Fig. 5a). All EDV/AAV-treated mice (8 out of 8) achieved complete responses (Fig. 5b). After rechallenge with OPM2 cells (5 × 10^6^) at day 35 after treatment, 3 out of 4 remitted mice maintained durable tumour control, indicating the establishment of functional persistence of the in vivo generated CAR T cells (Fig. 5b).

**Extended Data Fig. 10:**
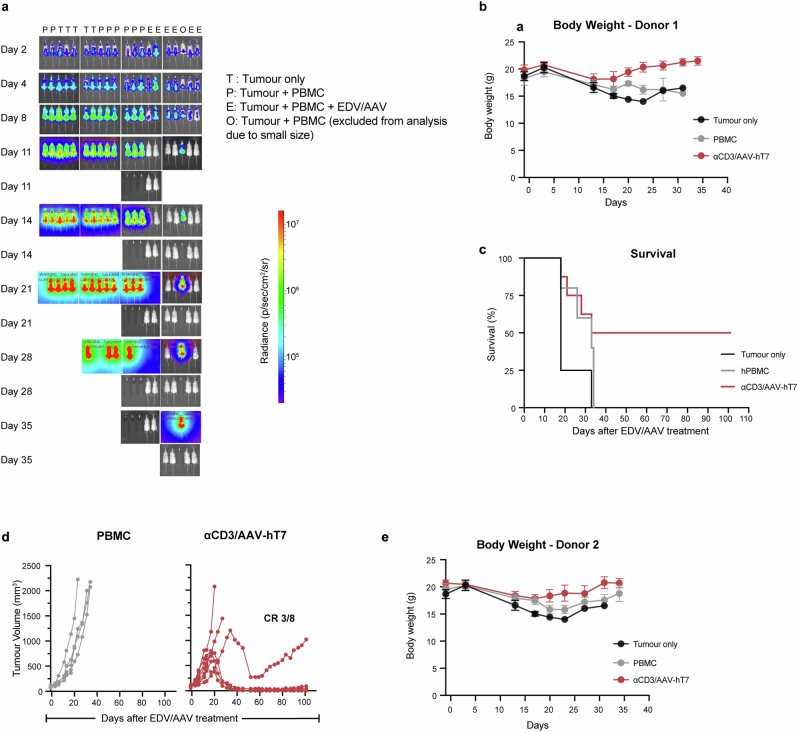
Related to main Fig. 5. **a**, Dorsal images from mice described in Fig. 5a. BLI is displayed as radiance (p/sec/cm^2^/sr). Days are indicated from time of PBS or EDV/AAV treatment. Day 11, 14, 21, 28 ans 35 are shown with and without mice that are saturating the signal. **b**, Total body weight from mice in Fig. 5c–e. **c**, Experimental conditions from Fig. 5c were repeated with an additional PBMC donor. Kaplan-Meier survival analysis in mice injected with MES-SA only (n = 4), MES-SA and PBMC (n = 5), MES-SA, PBMC and EDV/AAV targeting a aB7H3-CD28z-1XX (n = 6) CAR to *TRAC*. **d**, Tumour measurements in mice injected with MES-SA only (n = 4), MES-SA and PBMC (n = 5), MES-SA, PBMC and EDV/AAV targeting a aB7H3-CD28z-1XX (n = 8) CAR to *TRAC*. **e**, Total body weight from mice in **c**,**d**. Source data

We next extended this approach to solid tumours, a setting in which in vivo CAR T cell generation has not previously been demonstrated. We designed an anti-B7H3 CAR construct targeting an antigen broadly expressed across solid malignancies^44^, incorporating a CD28ζ−1XX signalling domain. NSG-MHC-I/II double-KO mice bearing subcutaneous MES-SA sarcomas were injected with PBMCs and treated with EDV/AAV vectors encoding the respective CARs (Fig. 5c). The anti-B7H3-CD28ζ−1XX CAR alone elicited complete responses in 5 out of 6 mice from one donor and in 3 out of 8 mice from a second donor (Fig. 5d,e and Extended Data Fig. 10a–d). These results demonstrate the use of EDV/AAV treatments beyond haematological malignancies.

Together, these data demonstrate site-specific in vivo CAR T cell generation using dually optimized AAV and EDV. This method can be used to successfully reprogram T cells in vivo to mount an antitumour cell therapy response against multiple haematological malignancies and a solid tumour model, which establishes in vivo *TRAC*-CAR T cell generation as a highly promising therapeutic platform.

## Discussion

Here we developed a method for site-specific integration of large DNA payloads in primary human T cells in vivo. By combining CD3-targeted Cas9-EDV and pre-existing neutralizing-antibody-resistant AAVs with enhanced T cell tropism, we generated therapeutic levels of *TRAC*-CAR T cells in vivo that control tumour growth in multiple tumour models.

Engineered LVVs are promising tools for CAR T cell therapies in vivo. However, constitutive promoters can drive CAR expression in any transduced cell, including tumour cells, which can downregulate the CAR target on the cell surface, leading to risks of antigen-negative relapse^21^. Constitutive promoters also drive CAR expression during viral packaging, incorporating CAR proteins within the viral envelope^45^. As a result, these particles can transduce tumours through CAR–antigen interactions, generating CAR-expressing tumour clones and increasing the risk of antigen-negative relapse^21^. These risks can be reduced using a lineage-specific promoter in the lentiviral design. Site-specific integration in vivo of a promoterless cassette within a lineage-specific promoter instead produces CAR T cell products with stable, predictable outcomes and T cell-specific CAR expression. The combined specificity of the CD3-targeted EDV, the more restricted tropism of AAV-hT7 and the *TRAC* promoter that is exclusively expressed in T cells provide a level of engineering specificity that is superior to existing methods. The combination of a cell-specific EDV, an evolved AAV and targeting a promoterless cassette to a lineage-specific promoter could be applied to other cell types. Furthermore, the methodology described in this study is not limited to CAR expression in T cells and could be applied to integrate TCR sequences or synthetic receptors that can be used to reprogram T cells directly in vivo.

We hypothesize that the efficacy of in vivo generated *TRAC*-CAR T cells arises from the uniform and regulated CAR expression conferred by site-specific integration at the *TRAC* locus. In this configuration, every successfully edited T cell expresses the CAR under the control of the endogenous *TRAC* promoter, resulting in consistent and functional surface expression across the population. By contrast, lentiviral transduction produces heterogeneous expression that depends on the MOI, a relationship readily observed when lentiviral particles are diluted ex vivo. Systemic delivery yields very low MOI, resulting in a mixed population in which many transduced cells express subthreshold levels of CAR that may be insufficient for optimal antitumor function. This model is consistent with our in vivo data: *TRAC-*CAR T cells expanded earlier and to much higher levels than lentiviral CAR T cells and displayed uniformly high and stable CAR surface expression compared with the lower and more variable expression observed in lentivirally engineered cells. Together, these findings support the notion that precise genomic targeting at the *TRAC* locus enables coordinated CAR expression across all engineered cells, thereby sustaining early activation, expansion and durable antitumour activity in vivo.

To achieve maximum in vivo editing efficiency, we used an optimized EDV using Cas9 tagged with four nuclear localization signals and an activating CD3 scFv—a feature that we found was necessary to achieve potent in vivo HDR. EDVs share similarities to other in vivo delivery vehicles derived from viral structures such as viral-like particles and enhanced viral-like particles^46–48^. To our knowledge, these systems have not been used to target T cells or insert large DNA payloads in vivo.

AAV6 is widely used for haematopoietic cell delivery in vitro, but is limited by serum neutralizing factors for in vivo use^49,50^. We engineered AAV-hT7 to improve in vivo generation of *TRAC*-CAR T cells using our evolution platform, which we designed to identify serum-resistant serotypes. We previously reported this strategy to identify a capsid for mouse T cells called Ark313 (ref. ^4^). Although AAV-hT7 displays resistance to pre-existing neutralizing antibodies, neutralizing antibodies can be generated against AAV-hT7 after injection, limiting the possibilities for redosing. We identify CD7 as an important host factor for AAV-hT7 uptake by human T cells. CD7 is expressed broadly across T cells, as well as mature NK cells^51,52^. High CD7 expression on T cells and its internalization after ligand binding probably contribute to efficient AAV-hT7 uptake by human T cells in vivo. In contrast to AAVs targeting CD4 or CD8 (refs. ^53,54^), the pan-T cell nature of CD7 expression offers a wide range of engineering capacity across T cell populations. We observe a strong effect of CD7 expression on AAV-hT7 uptake at lower MOI, which probably mimics the physiological situation as T cells encounter AAVs in vivo. Although our data show that CD7 expression affects AAV-hT7 uptake, it is important to note that transduction is not fully abolished after *CD7* KO. This probably indicates that some natural AAV uptake mechanisms remain intact, and that CD7 probably acts as a co-factor to induce AAV uptake. Nevertheless, on the basis of our in vitro experiments, it is likely that AAV-hT7 can be used to engineer CD7^+^ NK cells in vivo.

Other AAVs have been used to target T cells, including a synthetic CD8-targeting AAV for murine T cells^22^ or AAV9 in a clinical trial against HIV (NCT05144386). However, it remains unclear how effective these strategies are in the presence of serum or off-target delivery in the case of AAV9.

Finally, here we show that a two-vector system enables independent engineering for protein (Cas9–RNP EDV) and nuclear DNA delivery (AAV-HDRT). Although we are using HDR-mediated integration, other methods, such as PASTE^55^, PASSIGE^56^ and CAST^57^, have been developed recently for precise integration of large DNA payloads. We believe that our dual-vector system, which offers transient expression of tailored endonucleases combined with an efficient DNA template nuclear delivery, will benefit the application of these approaches.

Biodistribution analysis showed no significant differences between AAV6 and AAV-hT7 in this study. However, histopathological examination of humanized mice confirmed substantial human T cell infiltration in tissues, consistent with the expected distribution of human immune cells in this model. This suggests that AAV detection in peripheral tissues may reflect trafficking of human T cells rather than direct vector transduction of non-lymphoid compartments. Moreover, cytokine profiling in these humanized mice showed no systemic inflammatory response after EDV/AAV administration, in agreement with previous studies using T cell-targeted AAV variants such as Ark313 in vivo^58^. We acknowledge that these analyses were conducted in an immunodeficient humanized mouse model, which limits evaluation of biodistribution, immunogenicity and safety. Comprehensive assessment of vector tropism and systemic cytokine responses will require studies in larger, immunocompetent models such as non-human primates. These investigations represent an important next step towards fully characterizing the translational potential and safety profile of this in vivo CAR T cell engineering platform.

We demonstrate cell-specific insertion of large DNA payloads at a precise locus in vivo. Crucially, we also overcome major barriers to the therapeutic application of CAR T cell generation in patients, with implications for cell therapies without the need for leukapheresis and cell manufacturing. We are optimistic that this advance will have a substantial effect on the design and implementation of cell therapy trials and improve access to cutting-edge CAR T cell treatments for patients.

## Methods

### Ethics statement

Leukopaks from deidentified healthy donors with Institutional Review Board-approved consent forms and protocols were purchased from StemCell Technologies (200-0092). Residuals from leukoreduction chambers after Trima Apheresis from deidentified healthy donors with Institutional Review Board-approved consent forms and protocols were purchased from Vitalant.

All of the mice in this study were treated following a protocol (AN182757) approved by the UCSF Institutional Animal Care and Use Committee (IACUC).

### Plasmids

For transient GFP expression, a scAAV plasmid with a CMV enhancer chicken B-actin intron (CAG) promoter was used (scAAV-CAG-GFP, Addgene, 83279). A similar ssAAV (ssAAV-CAG-GFP) has been used for biodistribution studies (Addgene, 28014).

A plasmid encoding the HIV protein gag fused to Cas9 expressing four Nuclear Localization Signal (4×NLS) was used to package Cas9 into EDVs^59^. For integrating genes at the *TRAC* locus, we used the same homology arms and sgRNA sequence as previously described^1^. An EGFRt sequence was cloned in following a 1928z-1XX sequence^33^. To potentially improve nuclease activity when combined with a Cas9-containing EDV, a U6 promoter expressing the sgRNA for targeting *TRAC* was cloned into the plasmids upstream of the left homology arm (pAAV-U6/TRAC-TRAC-1928-1XX-P2A-EGFRt). The extracellular domain of a BCMA-targeting CAR was used to replace the CD19-binding sequence to generate a pAAV-U6/TRAC-TRAC-BCMA-1XX-P2A-EGFRt.

Plasmids for the B7H3 experiment were designed by cloning the scFv targeting B7H3 into a 28z-1XX CAR. The construct was flanked by the same homology arms used previously targeting *TRAC*.

To generate a GFP fusion at the *Clta* N terminus, the *GFP* gene was cloned into an AAV plasmid containing homology arms targeting the *CLTA* exon 1 start codon (pAAV-CLTA-GFP). The design homology arm design and sgRNA sequences were taken from a previous study^41^.

To produce lentiviruses delivering an anti-CD19 CAR, a second-generation lentivirus comprising a transfer plasmid, a packaging plasmid and an envelope plasmid were used. The exact CAR sequence from pAAV-U6/TRAC-TRAC-1928-1XX-P2A-EGFRt cloned into a lentiviral transfer plasmid, under an EF1-α promoter, was used. The AAV-hT7 capsid variant generated in this study has been made available at Addgene (252215).

### Cell lines

AAVs were packaged in HEK293T cells (ATCC, CRL-3216) or suspension-adapted HEK293 cells (Viral Production Cells 2.0, Gibco, A49784). Lentivirus were packaged in Lenti-X 293T cells (Takara, 632180). HEK293T and Lenti-X 293T cells were cultured in GlutaMAX DMEM (Gibco, 10566024) supplemented with FBS (10%, Corning, 35016CV), penicillin–streptomycin (100 U ml^–1^; Thermo Fisher Scientific, 15140122), sodium pyruvate (1 mM; Gibco, 11360070) and HEPES (10 mM; Corning, 25-060-CI). Suspension HEK293 cells were grown in viral production medium (Gibco, A4817901) supplemented with 20 mM GlutaMAX (Gibco, 35050061).

NALM6 parental lines and derivatives were cultured in RPMI 1640 (Gibco, 11875093) supplemented with FBS (10%), penicillin–streptomycin (100 U ml^−1^), sodium pyruvate (1 mM), HEPES (10 mM), β-mercaptoethanol (Gibco, 21985-023) and MEM non-essential amino acids (1×; Gibco, 11140050). OPM2-ffLuc-GFP cells were cultured in RPMI 1640 supplemented with penicillin–streptomycin (100 U ml^−1^) and FBS (10%). Raji (ATCC, CCL-86) cells were cultured in RPMI 1640 (Gibco, 11875093) supplemented with FBS (10%) and penicillin–streptomycin (100 U ml^−1^). JeKo1 (ATCC, CRL-3006) cells were cultured in RPMI 1640 (Gibco, 11875093) supplemented with FBS (20%) and penicillin–streptomycin (100 U ml^−1^). SupB15 (ATCC, CRL-1929) cells were cultured in Iscove’s modified Dulbecco’s medium with and supplemented with β-mercaptoethanol (Gibco, 21985-023), FBS (20%) and penicillin–streptomycin (100 U ml^–1^). MES-SA cells (ATCC, CRL-1976) were cultured in McCoy’s 5a modified medium (Gibco, 16600082) supplemented with 10% FBS and penicillin–streptomycin. Suspension HEK293 cells were maintained in an 8% CO_2_ incubator at 37 °C, on a 25 mm orbital shaker. All of the other cell lines were cultured under sterile conditions in a 5% CO_2_ incubator at 37 °C.

### Primary cell isolation and culture

#### PBMCs

PBMCs were used either fresh or from frozen aliquots. Apart from fresh PBMCs used for in vivo experiments described in Figs. 1i, 3a and Extended Data Fig. 7a, all PBMCs were sourced from Leukopaks (StemCell Technologies, 70500.1). Fresh Leukopaks were either directly processed for further cell purification or were frozen in aliquots.

For the experiments shown in Figs. 1i and 3a and Extended Data Fig. 7a, PBMCs were isolated from TRIMA residuals (Vitalant) using Lymphoprep reagent (StemCell Technologies, 07801) and SepMate-50 tubes (StemCell Technologies, 85450) according to the manufacturer’s protocol.

### T cells

Human T cells were isolated from frozen aliquots of PBMCs. After thawing, T lymphocytes were purified using the EasySep Human T cell isolation kit (StemCell Technologies, 17951) and activated with Dynabeads Human T Expander CD3/CD28 at a 1:1 bead-to-cell ratio (Gibco, 11141D) in X-VIVO 15 medium (Lonza, BP04-744Q) supplemented with human serum (5%, Gemini Bioproducts, 100-512), IL-7 (5 ng ml^−1^, Miltenyi Biotec, 130-095-367) and IL-15 (5 ng ml^−1^, Miltenyi Biotec, 130-095-760) at a density of 1 × 10^6^ cells per ml. Unless otherwise specified, beads were removed after 48 h, and T cells were maintained at 1 × 10^6^ cells per ml every 2 to 3 days by addition of fresh medium.

#### NK cells

NK cells were isolated from fresh PBMCs (TRIMA residual, Vitalant) by negative selection using the EasySep Human NK Cell Enrichment Kit (StemCell Technologies). Isolated NK cells were cultured at an initial density of 10^6^ cells per ml in NK MACS medium (Miltenyi) supplemented with human platelet lysate (5%, Elite Cell), penicillin–streptomycin (0.5%) and IL-2 (1,000 U ml^−1^, Peprotech), as previously described^60^. After an overnight rest period, cells were activated for 7 days with anti-CD2- and anti-NKp46-coated (Miltenyi) beads at a 1:2 bead-to-cell ratio.

#### Macrophages

Fresh PBMCs were obtained from StemCell Technologies (70500.1) and subjected to the EasySep Human CD14 positive selection kit II (StemCell Technologies, 17858) according to the manufacturer’s protocol. Then, 0.5 × 10^6^ isolated CD14 monocytes were seeded into 24-well plates for differentiation into human monocyte-derived macrophages in RPMI with 10% FBS, 1% penicillin–streptomycin, 1× GlutaMAX, 1× HEPES and 20 ng ml^−1^ recombinant human GM-CSF (PeproTech, 300-03) for 9 days.

#### HSCs

HSCs were isolated from G-CSF-mobilized peripheral blood of de-identified healthy donors and CD34 enriched and then cryopreserved (Fred Hutchinson Cancer Center, Hematopoietic Cell Procurement and Processing Core). Cryopreserved cells were thawed and incubated for 1 h at 37 °C in RPMI 1640 medium (Thermo Fisher Scientific) supplemented with 30% FBS, 1% penicillin–streptomycin, 10 μg ml^−1^ DNase I and 20 U ml^−1^ heparin. As previously described^61^, HSPCs were then cultured at 1 × 10^5^ cells per ml in StemSpan Serum-Free Expansion Medium II (StemCell Technologies) or Good Manufacturing Practice Stem Cell Growth Medium (SCGM, CellGenix) supplemented with a human cytokine cocktail (PeproTech) that included 100 ng ml^−1^ stem cell factor, 100 ng ml^−1^ thrombopoietin, 100 ng ml^−1^ FMS-like tyrosine kinase 3 ligand, 100 ng ml^−1^ interleukin-6, 20 mg ml^−1^ streptomycin and 20 U ml^−1^ penicillin.

### EDV and LVV production

#### Transfection

Lenti293T cells were seeded into packaging medium consisting of OptiMEM-GlutaMAX (Gibco, 51985034) supplemented 5% FBS and 1 mM sodium pyruvate. Cells were transfected the next day with the appropriate plasmids to produce EDVs or replicative-deficient LVV. A total of 60 μg of plasmids complexed with 181 μl of Lipofectamine 3000 and 165 μl p3000 were used per T225 flasks. EDVs were produced by transfecting producer cells with an envelope plasmid (env) and two packaging plasmids, one encoding a Gag–Cas9 fusion (Gag–Cas9), and one rescue packaging plasmid (PsPax2). A U6 promoter and sgRNA sequence was incorporated in both packaging vectors to generate EDV-carrying RNPs (Supplementary table 1). Env-to-gagCas9-psPax2 mass ratios were respectively 1:2:1 (v/v) were produced by transfecting producer cells with an *env* plasmid, a PsPax2 plasmid and a transfer plasmid encoding a 1928z-1XX sequence. The mass ratios of Env to psPax2 to transfer were 1:3:0.75 (ref. ^27^).

#### Particle pseudotyping

WT EDVs and LVVs were generated using the pMD2.G plasmid as the env plasmid. For T cell-targeted EDVs and LVVs, the pMD2.G plasmid was substituted with a construct encoding a mutated VSV-G glycoprotein (K47Q, R354A) fused through a P2A linker to an scFv specific for CD3.

#### Collection and isolation

The medium was replaced after 6 h with fresh packaging medium supplemented with 0.2% (v/v) ViralBoost (Alstem, VB100). LVVs and EDVs were collected 48 h after transfection. Cell debris was pelleted by centrifugation at 2,000*g* for 10 min. The supernatant was clarified through a 0.45 μm filter before purification by ultracentrifugation on a sucrose cushion (50 mM Tris-HCl, 100 mM NaCl, 0.5 mM EDTA, 20 w/v% sucrose, pH 7.4). The supernatants were spun down at 91,000*g* for 90 min. Pellets were resuspended in ice-cold PBS and immediately stored at −80 °C.

#### Particle titration

EDVs were titred by measuring the sgRNA concentration in the particles, as previous studies showed that the number of sgRNA equates to the number of functional Cas9 RNPs inside the EDVs^27^. The sgRNA concentration was determined using quantitative PCR with reverse transcription (RT–qPCR). EDVs were diluted tenfold in DirectDetect buffer (Zymo Research, R1400) according to the manufacturer’s instructions. For RT–qPCR, custom TaqMan small RNA assays were designed to detect the sgRNA sequence (Thermo Fisher Scientific, CTZTEYN (for *CLTA* sgRNA) and CTCE4RX (for *TRAC* sgRNA)). Synthetic sgRNA sequences with the appropriate spacers were used as standards. We added 4 μl of samples or standards to 6 μl of RT–qPCR mastermix (1× Luna Luna Universal One-Step RT–qPCR mix with 0.25× RT primer and 1× small RNA assay probes from TaqMan small RNA assay kit in nuclease-free water) in 384-well plates. RT–qPCR was performed on the QuantStudio 5 Real-Time PCR System (Thermo Fisher Scientific) using the following parameters: carryover prevention (25 °C, 30 s), reverse transcription (55 °C, 15 min), initial denaturation (95 °C, 1 min) and 45 cycles of denaturation (95 °C, 10 s), extension (60 °C, 60 s) with a plate read.

For the comparative study between the combined EDV and AAV treatment and the LVV treatments, two normalization methods were used. The first normalization was based on the number of anti CD3-pseudotyped particles (LVV and EDV) injected. Physical titres of LVV and EDV preparations were measured through ELISA-based p24 quantification (Lenti-X p24 Rapid Titer Kit, Takara, 632200) according to the manufacturer instructions. The total p24 amount per EDV dose was calculated based on the sgRNA titres (here, 5 × 10^11^ sgRNA per mouse) and a matching p24 amount of LVV was injected. The mice injected with these samples are referred to as the LVV^high^ dose group.

Functional normalization was also performed. Transducing units (TU) were measured based on the ability to generate CAR T cells in vitro, according to the formula below.

$$\mathrm{TU}\,\mathrm{per}\,\mathrm{ml}=\frac{\mathrm{number}\,\mathrm{of}\,\mathrm{cells}\,\mathrm{transduced}\times \mathrm{percentage}\,\mathrm{of}\,{\mathrm{CAR}}^{+}\,\mathrm{cells}}{\mathrm{volume}\,\mathrm{added}\times \mathrm{dilution}\,\mathrm{factor}}$$

As AAVs have been found to be non-limiting in in vitro settings, a fixed amount of AAV was used with decreasing doses of EDVs to calculate the total TU_EDV_ per preparation. LVVs were also used in decreasing doses to calculate total TU_LVV_. The sgRNA dose of EDV was converted into TU_EDV_ and a similar TU_LVV_ dose was injected. The mice injected with these samples are referred to as the LVV^low^ dose group.

According to manufacturer guidance and previous work^27^, 5 × 10^11^ sgRNA molecules are the equivalent to approximately 1.1 × 10^11^ EDV particles or 8.8 µg of p24. Approximatively three times less LVV was injected for the functional normalization.

### AAV production and quantification

AAV2-ITR-containing plasmids were used to package vector genomes into various AAV capsids by transfection of HEK293T cells with adenovirus helper and AAV Rep-Cap plasmids using polyethyleneimine (Polysciences, 23966). For small-scale production, HEK293T cells were seeded into 150 mm dishes, and a total of 20 μg of plasmid complexed with 200 μl PEI was used per dish. The mass ratio of cargo:Rep-Cap:helper was 1:1.3:1.8.

Cell pellets were collected 72 h after transfection and resuspended in AAV lysis buffer (50 mM Tris, 150 mM NaCl) before being lysed by three rounds of rapid freeze–thawing. Polyethylene glycol (PEG) was used for precipitation of supernatants. Crude lysates and pelleted supernatants were combined before downstream purification.

For large-scale production, suspension-adapted HEK293 cells were transfected using the VirusGEN AAV Transfection Kit (Mirus, MIR 6750) according to the manufacturer’s recommendations. A total of 2 μg of plasmid were used per millilitre of cell culture, while keeping the same mass ratios. Producer cells were lysed 72 h after transduction by chemical disruption (AAV Lysis Buffer, Gibco, A50520) and crude lysates were concentrated by PEG precipitation.

AAV-containing solutions were incubated for 1 h at 37 °C with 25 U ml^–1^ benzonase (Millipore Sigma, 70-664-3). Solutions were clarified through centrifugation and AAV vectors were further purified using iodixanol (OptiPrep, StemCell Technologies, 07820) gradient ultracentrifugation.

AAV vector titres were determined by qPCR on DNase-I-treated (NEB, B0303S), proteinase-K-digested (Qiagen, 1114886) AAV samples after purification, using primers targeting the viral genome. qPCR was performed with PowerUp SYBR Green Master Mix (Applied Biosystems, A25918) on a StepOnePlus Real-Time PCR System (Applied Biosystems, 4376600). Relative quantity was estimated by comparison to a serial dilution of a vector plasmid standard of known concentration.

### AAV capsid library generation and evolution

The AAV6 capsid library was generated by performing saturation mutagenesis of seven residues in the VR-IV region as reported previously^62^. In brief, to generate the library, an overlap extension PCR was performed using two amplicons amplified from a modified AAV6 backbone containing seven tandem stop codons replacing the randomized region (amino acids 454–460 VP1 numbering) to prevent potential amplification of the WT sequence. The first amplicon consisted of 34 bp of the AAV6 cap immediately 5′ of the randomized region, the randomized region and the AAV6 cap up to the Sbf1 site 3′ of the randomized region. The second amplicon consisted of the AAV6 cap starting from the BsiWi site 5′ of the randomized region up to the 34 bp of the AAV6 cap immediately 5′ of the randomized region. The two resulting amplicons were combined in an equimolar ratio in a second PCR for overlap extension. The final assembled amplicon was digested using BsiWI-HF (NEB, R3553S) and SbfI-HF (NEB, R3642S) and ligated into the pITR2-Rep2-dead(GFP)Cap6 backbone using T4 DNA ligase (NEB, M0202S). The pITR2-Rep2-dead(GFP)Cap6contains AAV2 ITRs and Rep along with the AAV6 Cap gene interrupted by a filler sequence derived from GFP inserted out of frame into the cognate BsiWI and SbfI site to eliminate any potential WT AAV6 from the library ligation. The ligation products were concentrated and purified by ethanol precipitation. Purified products were electroporated into DH10B ElectroMax cells (Invitrogen, 18290015) and directly plated onto multiple 5,245-mm^2^ bioassay dishes (Corning, 431111) with LB/ampicillin agar to maintain library diversity. Plasmid DNA from AAV6 capsid libraries was purified from pooled colonies grown on LB agar plates with ampicillin using the ZymoPURE II plasmid maxiprep kit (Zymo Research, D4203).

AAV6 capsid libraries were produced by co-transfection of adherent HEK293T cells with adenovirus helper plasmid (pXX680,49 19.25 μg per plate) and the Rep Cap plasmid library (8.75 μg per plate) mixed in a 3:1 ratio with PEI MAX (Polysciences, 24765-1). Viral medium was collected at day 4 and 6, and the cell pellet at day 6. The medium was PEG precipitated, and the cell pellet was lysed through chemical disruption. AAVs were purified as described above.

Library evolution was performed by transducing 48-h-activated T cells with the capsid library at a MOI of 1 × 10^4^. To evolve the library, primary human T cells were isolated and activated with CD3/CD28 beads and recombinant IL-7/IL-15 and then co-cultured with the capsid library at a low MOI of 1 × 10^4^. Cells were then washed twice with PBS to remove any unbound AAV, and cellular and viral DNA was extracted from cells using the IBI genomic DNA extraction kit (IBI Scientific, IB47280). The Cap region was amplified by PCR before being digested and ligated back into the pITR2-Rep2-dead(GFP)Cap6 backbone to generate the next-round library.

A total of three rounds of evolution was performed to generate an evolved library. Each round of library was performed on two different donors, for a total of six different donors used.

### AAV6 capsid library sequencing and analysis

Parental and evolved libraries were processed for Illumina NovaSeq sequencing. Parental and evolved libraries were each treated with DNase I and purified by iodixanol gradient centrifugation. To dissociate the capsid, virus was heated in a PCR tube (95 °C, 15 min) with Tween-20, which prohibits capsid reassembly that would interfere with amplification.

Round-1 PCR was performed with defined primer sets (Supplementary Table 1) for 18 cycles using Q5 polymerase (NEB, M0492S), and amplicons were PCR-purified (IBI Scientific, IB47010). In round 2, indices for demultiplexing and the P5 and P7 flow cell adaptor sequences were added in a 15-cycle PCR, and amplicons were run on and purified from a 1% agarose gel. The amplicon band was gel-purified, amplicon quality was verified using a Bioanalyzer (Agilent) and concentrations were quantified by Qubit (Invitrogen). Libraries were prepared using the Illumina NovaSeq 6000 S-Prime reagent kit (300 cycles, Illumina, 20028312) according to the manufacturer-provided instructions and sequenced by Illumina NovaSeq.

Demultiplexed reads were analysed using an in-house Perl script as done in another context previously^63^. Reads were probed for the nucleotide sequences corresponding to the library region, and the occurrence of each nucleotide sequence was counted and ranked. These sequences were converted to amino acid sequences and pooled by like-sequence, counted and ordered by percentage rank. A second Perl script was used to calculate enrichment between the evolved library and parental library as done previously^63^. Data in Supplementary Table 2. To generate the amino acid position-specific scoring matrix, sequences that were above 0.1% of the reads of the evolved library and enriched more than 100-fold from the parental library (34 sequences) were selected and run through PSSMSearch (http://slim.icr.ac.uk/pssmsearch/).

### Combined EDV + AAV transduction

Unless otherwise specified, combined EDV + AAV transduction of T cells was performed as follows. 48-h-activated T cells were seeded at 1 × 10^6^ cells per ml in T cell medium, and particles were added at a specified MOI. The volume of EDV/AAV added did not exceed 20% of the culture volume. After incubating the culture overnight, the AAV-containing medium was exchanged for fresh medium, and T cells were subsequently cultured in standard conditions.

### Genome-wide CRISPR screens identify AAVs host factors

#### Genome-wide CRISPR–Cas9 screening

A genome-wide sgRNA KO library targeting 19,114 genes (comprising 76,441 sgRNAs) was sourced from Addgene (Brunello Library, 73178) and amplified according to the guidelines for maintaining library representation^64^. Viral packaging of the library in lentivirus was conducted as previously described. Screens were performed with cells isolated from two healthy donors. T cells were obtained from thawed PBMC aliquots and activated using Dynabeads. Then, 36 h after activation, T cells were transduced with the lentiviral library at an MOI of approximately 0.3. The lentiviral titre was predetermined using T cells from the same donors. For this experiment, integration rates were measured at 40–45% after puromycin selection. After a further 36 h, beads were removed and Cas9 protein was introduced via nucleofection. Nucleofection was performed using the P3 Primary Cell 96-well Nucleofector Kit (Lonza V4SP-3096) on the Amaxa 4D Nucleofector unit (Lonza, AAF-1002B). Cells were resuspended at 1 × 10^8^ per ml in P3 buffer and Cas9 protein (40 μM stock) was added to the cells at a 1:20 ratio and then electroporated using pulse code EH-115.

Cells recovered for 2 days, then underwent puromycin selection at 2 µg ml^−1^ (Gibco A1113803) for another 2 days. After selection, cells were washed twice, cultured for two more days, and restimulated with Dynabeads for 48 h at a 1:10 beads-to-cell ratio to enhance transcriptional activity while limiting activation-induced cell death.

Cells were transduced at an MOI of 5 × 10^3^ in serum-free conditions. After overnight transduction, cells were washed, and serum was reintroduced by replenishing cells with fresh medium. Then, 48 h after AAV transduction, cells were prepared for sorting by staining with 7-AAD live/dead reagent (eBioscience, 00-6993-50), followed by fixation in 4% formaldehyde in PBS (15 min, 4 °C) at a concentration of 10^7^ cells per ml. Fixed cells in FACS buffer were sorted into four bins based on GFP expression. More than 4.5 × 10^7^ cells per replicate were sorted at the UCSF Parnassus Flow Cytometry Core (PFCC).

After cell sorting, genomic DNA extraction was performed according to established protocols^65^. sgRNA barcodes underwent PCR amplification utilizing Ex Taq DNA Polymerase (Takara Bio) through 28 thermal cycles, followed by amplicon purification with SPRIselect Beads (Beckman Coulter). Quality assessment was conducted using D1000 ScreenTape assay on a TapeStation system (Agilent). For sequencing preparation, P7 and P5 primers for lentiGuide compatibility (generating 354-nucleotide products). PCR amplification of sgRNA sequences was performed using standard protocols (https://portals.broadinstitute.org/gpp/public/) with P5/P7 primers obtained from IDT.

Library pooling preceded sequencing on a NovaSeq X platform (300 × 151 × 12 × 24 × 151 configuration) across two lanes at UCSF’s Center for Advanced Technology. Post-sequencing FASTQ files underwent processing and analysis using MAGeCK v.0.5.9.5. Data visualization was performed using volcano plots with the log-transformed fold change on the* x* axis (negative values indicating GFP-low bin enrichment) and −log_10_[*P*] on the *y* axis, representing the inverse error probability where higher values correspond to increased statistical confidence. Data are provided in Supplementary Table 3.

#### Individual validation

To validate the function of genes identified in the genome-wide screens, individual gene knockouts were performed by delivering Cas9–RNP complexes. T cells activated for 48 h were resuspended at a concentration of 1 × 10^8^ cells per ml in P3 buffer. RNP complexes were generated by incubating Cas9 protein (40 μM stock) with targeting sgRNAs (80 μM stock) at a molar ratio of 2:1 for 15 min at 37 °C. For each experiment, 3 μl of RNP complexes were added to 20 μl of resuspended T cells. Alternatively, *CD7-*KO T cells were generated using base-editing by adding 1 µg mRNA encoding an adenine base editor and 100 pmol CD7-targeted sgRNA to 20 µl resuspended T cells. Electroporations were conducted using pulse code EH-115.

Edited T cells were cultured for 6–8 days, reactivated with Dynabeads at a 1:10 beads-to-cell ratio for 48 h, then transduced with various AAVs carrying sc-CAG-GFP at specified MOIs. The medium was changed the next day, and GFP expression was assessed using flow cytometry 48 h after transduction.

### Animal work

All mice in this study were treated following a protocol approved by the UCSF Institutional Animal Care and Use Committee (IACUC) and were housed under a 12 h–12 h light–dark cycle with food and water available ad libitum. Unless specifically mentioned, all procedures were performed in a BSL-1 room. NSG (005557) and NSG-MHCI/II double-KO (025216) mice were acquired from the Jackson Laboratory. Only female mice were used in this study.

#### Humanization

Unless specifically mentioned, all humanization was performed using frozen aliquots of PBMCs. PBMCs were thawed on the day of injection, washed, counted and kept in ice-cold RPMI medium before injection. NSG-MHCI/II double-KO mice (aged 7–12 weeks) were injected i.v. with 1 × 10^7^ of thawed PBMCs.

#### Organ collection for phenotyping

Spleens were collected, crushed in FACS buffer, strained and then treated with 1 ml ACK lysing buffer (Quality Biological, 118-156-101) for 2 min. The lysing process was quenched by adding 20 ml FACS buffer, the cells were then strained and resuspended in FACS buffer and stained for flow cytometry.

Bone marrow was collected by crushing the hind legs (femur and tibia) using a mortar and pestle in FACS buffer. The crushed bones marrows were then strained and treated with 1 ml ACK lysing buffer (Quality Biological, 118-156-101) for 2 min. The lysing process was quenched by adding 20 ml FACS buffer, the cells were then strained and resuspended in FACS buffer and stained for flow cytometry.

Circulating blood was collected through submandibular cheek bleeding. Blood samples were collected in EDTA-coated tubes. Red blood cells were lysed with RBC lysis buffer (BioLegend, 420302) for 15 min at room temperature.

#### B cell depletion models

For experiments with MHC-intact mice, 8-week-old NSG mice were injected i.v. with 1 × 10^7^ frozen PBMCs. EDVs and AAVs were injected 10 days later. For experiments with MHC-KO mice, NSG-MHCI/II double-KO mice were engrafted i.p. with 2 × 10^7^ fresh human PBMCs isolated from Trima residuals, followed by EDV and AAV injection 5 days later.

For all experiments, mice were injected i.v. with a mixture of AAV (1 × 10^12^ vg per mouse) and EDV (2.5−5 × 10^11^ sgRNAs per mouse) in PBS and euthanized for organ collection 14 days after EDV/AAV injection.

### Tumour models

#### Experimental scheduling

Across all models and to support tumour engraftment, mice were first injected with a given dose of tumour cells before humanization. Humanization was performed either i.p. or i.v., at the indicated time. Mice were injected at the indicated time with a mixture of AAV (1 × 10^12^ vg per mouse) and EDV (2.5−5 × 10^11^ sgRNA per mouse) in ice-cold PBS. Only MHC-I/II double-KO mice were used for tumour challenges.

#### Tumour measurement and end points

Mice were humanely euthanized at an IACUC-approved humane end point, including respiratory distress, hunched posture, body condition score of 2 or less, 15% weight loss, impaired or decreased mobility or neurological signs that interfere with normal function.

For haematological malignancies (NALM6 and OPM2), tumour burden was monitored at regular intervals by BLI using the Xenogen IVIS Imaging System (Xenogen) with Living Image software (Xenogen) for acquisition of imaging datasets. Both dorsal and ventral images were obtained for each animal. The dorsal and ventral signals were separately quantified through region of interest (ROI). The resulting signal summations (in units of photons per s) were normalized to the ROI area so that all measurements are given in photons per s per cm^2^.

For solid tumour challenge, the tumour volume was measured using callipers by first determining the length as the longest dimension and the width as the shortest perpendicular dimension of the tumour. Tumour volume was calculated according to the standard formula below.

$$\frac{1}{2}\times \mathrm{length}\times {\mathrm{width}}^{2}$$

#### B-ALL experiments

NSG MHC-I/II double-KO (aged 7–9 weeks) mice were purchased from Jackson Laboratories. Apart from the experiment described in Fig. 1i–j, mice received 5 × 10^5^ NALM6 ffLuc-GFP by i.v. injection and were humanized 3 days later by i.v. injection of 1 × 10^7^ of PBMCs from frozen stocks. For the experiment in Fig. 1i–j, NSG-MHCI/II double-KO mice were i.v. injected with 2.5 × 10^5^ NALM6-FFLuc-GFP, followed 3 days later by i.p. injection with 2 × 10^7^ fresh PBMCs.

In all cases, EDV and AAV were injected the day after PBMC injection. Mice were injected either i.p. or i.v. with a mixture of AAV (1 × 10^12^ vg per mouse) and EDV (2.5–5 × 10^11^ sgRNAs per mouse).

For the experiment in Fig. 4c–j, LVV injection was performed at the same time as EDV and AAVs. Injected doses of LVV were normalized to EDV/AAV treatment based on the titres (the quantification methods are described above). Two normalization methods were used. An equivalent number of EDV and LVVs was injected, based on the quantification of particles in the two viral preparations (LVV^high^). Volumes were also normalized based on the functionality, so that an equivalent TU was injected between the EDV/AAV and the second LVV condition (LVV^low^). For this experiment, the EDV-to-LVV volume ratio was 1:0.7 for the first method, and 1:0.2 for the latter.

Ex vivo manufactured cells were generated from T cells isolated from the same PBMCs aliquots. T cells were isolated and activated for 2 days. Cells were left untreated, treated with a WT pseudotyped LVV delivering a CD19-28z-1XX cargo, or electroporated with TRAC targeted sgRNAs and Cas9 protein. Electroporated cells were either left for recovery or transduced with an AAV delivering an HDRT to precisely knock-in the same CAR CD19-29z-1xx at the *TRAC* locus.

CAR expression was measured by flow cytometry 5 days after treatment, and then again before freezing at day 10 of expansion. Cells were counted and frozen on the CryoStor CS10 (StemCell Technologies, 100-1061). T cells were thawed on the day of injection, washed in RPMI and injected i.v. in non-humanized age-matched mice.

#### Multiple myeloma model

MHC-I/II double-KO mice (aged 8 weeks) were purchased from Jackson Laboratories, mice were injected i.v. with 1 × 10^6^ OPM2 ffLuc-GFP, then humanized i.v. 5 days later with 1 × 10^7^ PBMCs from frozen vials. EDV/AAV treatment was performed 5 days after humanization through i.v. injection.

#### Sarcoma model

MHC-I/II double-KO mice (aged 8 weeks) were obtained from Jackson Laboratories. MES-SA cells (4 × 10^6^) were suspended 1:1 in PBS and Matrigel and injected subcutaneously into the flank of immunocompromised mice. Matrigel supported cell viability and tumour establishment. Tumour growth was monitored regularly.

#### TCR-seq analysis

To isolate in vivo engineered CAR T cells, splenocytes were labelled with anti-EGFR-biotin (BioLegend, 352934) and isolated using anti-biotin MicroBeads (Miltenyi Biotec, 130-090-485) on LS columns (Miltenyi Biotec, 130-042-401). T cell clonality was analysed on RNA from isolated CAR T cells using SMART Human TCR a/b Profiling Kit (Takara Bio, 634780). Samples were pooled and sequenced on the Illumina MiSeq system using 2 × 300 bp paired-end chemistry and the MiSeq Reagent Kit v.3 (MS-102-3003, Illumina). FASTQ files were analysed using MiXCR to identify TCR clonotypes. Clonotypes were defined by unique CDR3 amino acid sequences combined with V and J gene assignments, and clonotypes with fewer than two reads were excluded from analysis. Clonality metrics and visualization were performed using custom Python scripts.

#### Biodistribution

For biodistribution studies, NSG-MHCI/II double-KO mice (aged 7 weeks) were first humanized with frozen PBMCs (1 × 10^7^ per mouse). Then, 10 days later, mice were injected i.v. with AAV6 or 312 delivering a ssAAV-CAG-GFP. After 1 week, mice were euthanized and transcardial perfusion was performed, then organs were collected and snap-frozen in liquid nitrogen. DNA extractions were performed using the PureLink Genomic DNA Mini kit (Invitrogen, K182001). Eluted DNAs were further cleaned and concentrated using NA Clean & Concentrator-5 Kit (Zymo, 1159U31).

Viral genomes were quantified by qPCR targeting the *GFP* gene, and the total copies were normalized to the DNA input.

#### Cytokine quantification in vivo

NSG-MHCI/II double-KO mice (aged 9 weeks) were humanized with frozen PBMCs (1 × 10^7^ per mouse). Ten days later, mice were i.v. injected with different AAVs (1 × 10^12^ per mouse), EDVs (5 × 10^11^ per mice) or a combination of both. Mice were euthanized at the indicated times and blood was immediately collected through cardiac puncture in non-treated tubes. Blood samples were allowed to clot, and the resulting serum was diluted 1:2 in PBS, stored at −80 °C, and shipped for analysis. Serum contents were analysed for a panel of 41 human and mouse cytokines (Eve Technologies, HUMU41)

### Flow cytometry

Cells were stained to distinguish dead cells in 100 μl PBS for 30 min at room temperature using either Zombie Violet (BioLegend, 423114), Zombie NIR (BioLegend, 423105) or Ghost Dye Red 780 (Tonbo, 13-0865-T100).

Cells were stained in 100 μl FACS buffer (2% FBS and 1 mM EDTA in PBS) for 30 min at room temperature using the following reagents: anti-TCRa/b AF488 (BioLegend, 306712), anti-TCRa/b BV421 (BioLegend, 344646), anti-G4S AF647 (Cell Signaling Technologies, 69782), anti-CD45 PE (BioLegend, 368510), anti-CD25 BV711 (BioLegend, 356138), anti-CD69 PE (BioLegend, 310906), anti-CD4 BUV395 (BioLegend, 583550), anti-CD8 BV421 (BD, 568217), anti-CD8 BV711 (BioLegend, 344734), anti-CD8 PE-Cy7 (Invitrogen 25-0087-42), anti-EGFR AF488 (BioLegend, 352908), anti-EGFR BV711 (BioLegend, 352290), anti-CD19 BUV737 (BD, 612756), anti-CD3 BUV395 (BD, 563546) and anti-CD56 BV711 (BioLegend, 318336).

For quantification of cells during flow cytometry, 50 μl of CountBright Absolute Counting Beads (Invitrogen, C36950) was added to each sample according to the manufacturer’s protocol.

### Spectral flow cytometry

#### Preparation of reference controls for spectral flow cytometry

For determining optimal controls for unmixing, reference controls for each marker were prepared on Ultra Comp eBeads Plus (Invitrogen 01-3333-42), Slingshot HyParComp (Slingshot SSB-20-A) and cells (1 million human PBMCs), except for LIVE/DEAD blue. Beads were stained with individual antibodies at a dilution of 1:100. Cells were stained with individual antibodies using the same concentration used in the surface and intracellular stain master mixes. Surface markers were diluted in FACS buffer and intracellular markers were diluted in permeabilization buffer. All of the reference controls underwent the same protocol as the fully stained samples, including incubation/staining times, washes and fixation/permeabilization steps. The same antibody lot was used for both reference controls and full-stained samples. For LIVE/DEAD blue staining, 1 million cells were heat killed at 65 °C for 15 min. Half of the cells were stained with LIVE/DEAD blue under the same conditions as full-stain samples (50 µl staining volume in PBS, 25 min at room temperature, protected from light), washed, then mixed with the remaining unstained dead cells. An unstained human PBMC sample was used in the reference group as a universal negative for the CD45-PE single-cell reference control. Reference controls were acquired on a five-laser Aurora spectral flow cytometer (Cytek Biosciences).

Data were unmixed using SpectroFlo v.3.3 software (Cytek Biosciences). Unmixing quality was assessed using bead versus cell controls and optimal reference controls were chosen as described in a previous study^66^. Moreover, the unstained reference control was used for unmixing with autofluorescence extraction. The resulting unmixed .fcs files were analysed using manual gating in FlowJo v.10.10 software (BD Biosciences). Panel performance was further assessed by generating NxN plots and manually inspecting marker expression in CD4^+^ and CD8^+^ T cells. Supplementary Table 2 reports the single stain reference control that was used for each marker in the panel.

#### Spectral flow staining and data acquisition

In total, 2 million isolated splenocytes were stained for each sample. Moreover, 2 million splenocytes were taken from one sample to serve as a group-specific unstained control for unmixing. To block Fc receptor binding, cells were incubated with FcR blocking reagent, mouse (Miltenyi 130-092-575) for 30 min at 4 °C. LIVE/DEAD fixable blue (Invitrogen, L34961) viability stain was reconstituted in 50 µl of DMSO and serially diluted 1:1,000 in PBS. Viability stain was added to all full-stain sample cells in a 50 µl staining volume and incubated at room temperature for 25 min protected from light. Cells were washed once with PBS and once with FACS buffer before proceeding to surface staining. To make the surface antibody master mix, antibodies conjugated to fluorophores excited by ultraviolet (UV), violet and blue lasers were added first into BD brilliant buffer (BD 00-4409-42). The remaining surface antibodies excited by yellow-green and red lasers were then added, followed by FACS buffer. Then, 50 µl of surface master mix was added to each well except for the unstained controls, to which 50 µl of FACS buffer was added. Cells were incubated with surface master mix at room temperature for 25 min protected from light, then washed twice with FACS buffer. Cells were fixed and permeabilized using the FOXP3/transcription factor staining kit (eBioscience 00-5523-00) at room temperature for 1 h protected from light (200 µl per well). The samples were washed once in a permeabilization buffer (full-stain samples) or FACS buffer (unstained sample). Unstained cells were resuspended in FACS buffer and full-stain samples were resuspended in permeabilization buffer and kept overnight at 4 °C protected from light. The next day, the intracellular antibody master mix was prepared by first adding ultraviolet, violet and blue excited fluorophores to BD brilliant buffer (BD 00-4409-42). The remaining intracellular antibodies excited by yellow–green and red lasers were then added, followed by a permeabilization buffer. Then, 50 µl of intracellular master mix was added to each well and cells were incubated for 1 h at 4 °C protected from light. After staining, cells were washed with permeabilization buffer then FACS buffer and resuspended in FACS buffer. Antibody information is provided in Supplementary Table 4.

Samples were acquired on a five-laser Aurora spectral flow cytometer (Cytek Biosciences). The reference control data were used within 1 week of acquiring by choosing ‘duplicate experiment with reference controls’ in the SpectroFlo v.3.3 software (Cytek Biosciences). In addition to acquiring the full-stain samples, a group-specific unstained control was recorded for unmixing with autofluorescence extraction of the full-stain samples. The samples were unmixed using the same single-stain reference controls and the same gating of positive and negative populations that was used when generating the single-stain controls. Full-stain samples were checked for unmixing errors using NxN plots after unmixing. The resulting unmixed .fcs files were analysed using manual gating in FlowJo v.10.10 software (BD Biosciences).

### Editing frequency analysis by dPCR

Between 2 and 4 days after editing, cells were collected and QuickExtract DNA extraction solution (Epicentre) was used to collect genomic DNA. In some cases, extracted genomic DNA was concentrated using SPRIselect (Beckman Coulter). The genomic DNA was then digested using HindIII-HF according to the manufacturer’s instructions (New England Biolabs). The percentage of targeted alleles within a cell population was measured using Qiagen’s QIAcuity One 5-Plex Digital PCR System and QIAcuity Software Suit (v.2.5.0.1; Qiagen) using the following reaction mixture: 1–4 μl of digested genomic DNA input, 3 μl of QIAcuity Probe PCR Master Mix (Qiagen), primer/probes (0.8 µM; Integrated DNA Technologies) and volume was brought up to 12 μl with H_2_O. The sample reaction mixture was then added to QIAcuity Nanoplate 8.5 K 24-well (Qiagen). QIAcuity dPCR (Qiagen) settings were as follows: 95 °C (10 min), 95 °C (15 s), 59.6 °C (30 s), and return to step-two ×40–50 cycles. Analysis of droplet samples was performed using the QIAcuity Software Suit (Qiagen). To determine percentages of alleles targeted, the numbers of Poisson-corrected integrant copies per ml was divided by the numbers of reference DNA copies per ml. The following primers and 6-FAM/ZEN/IBFQ-labelled hydrolysis probes were purchased as custom-designed PrimeTime qPCR assays from Integrated DNA Technologies (Supplementary Table 1).

### Statistics and reproducibility

The statistical methods and the number of mice or donors are indicated in figure legends for each experiment. In vivo experiments were performed with multiple donors or preceded by pilot experiments.

### Reporting summary

Further information on research design is available in the Nature Portfolio Reporting Summary linked to this article.

## Online content

Any methods, additional references, Nature Portfolio reporting summaries, source data, extended data, supplementary information, acknowledgements, peer review information; details of author contributions and competing interests; and statements of data and code availability are available at 10.1038/s41586-026-10235-x.

## Supplementary information

Supplementary Figures Supplementary Figs. 1–6: gating strategies for the figures and extended data figures.

Reporting Summary

Supplementary Table 1 Oligonucleotide sequences.

Supplementary Table 2 Data from AAV evolution.

Supplementary Table 3 MAGeCK data from GW CRISPR screen. *P* values were determined using MAGeCK RRA one-sided tests and methods.

Supplementary Table 4 Antibodies used for spectral flow.

## Source data

Source Data Fig. 1

Source Data Fig. 2

Source Data Fig. 3

Source Data Fig. 4

Source Data Fig. 5

Source Data Extended Data Fig. 1

Source Data Extended Data Fig. 2

Source Data Extended Data Fig. 3

Source Data Extended Data Fig. 4

Source Data Extended Data Fig. 5

Source Data Extended Data Fig. 6

Source Data Extended Data Fig. 7

Source Data Extended Data Fig. 8

Source Data Extended Data Fig. 9

Source Data Extended Data Fig. 10

## Data Availability

The data for the genome-wide screen described in this study have been deposited in the NCBI BioProject database under accession number PRJNA1412486. Source data are provided with this paper.

## Code availability

All analysis code is available at GitHub (https://github.com/charwang0440/nature_in_vivo_ht7) and archived on Zenodo^67^ (10.5281/zenodo.17372997). The repository includes MAGeCK-based CRISPR screen analysis and MiXCR-based TCR analysis pipelines with documentation and environments.
