# Supplementary material for: In vivo site-specific engineering to reprogram T cells
Source: Nature. 2026 Mar 18;652(8110):712–21. doi: 10.1038/s41586-026-10235-x (PMC13083257; doi:10.1038/s41586-026-10235-x)

nature portfolio

https://doi.org/10.1038/s41586-026-10235-x

Supplementary information

In vivo site-specific engineering to reprogram T cells

In the format provided by the authors and unedited

Supplementary Figure 1: Gating strategy for Figure 1d

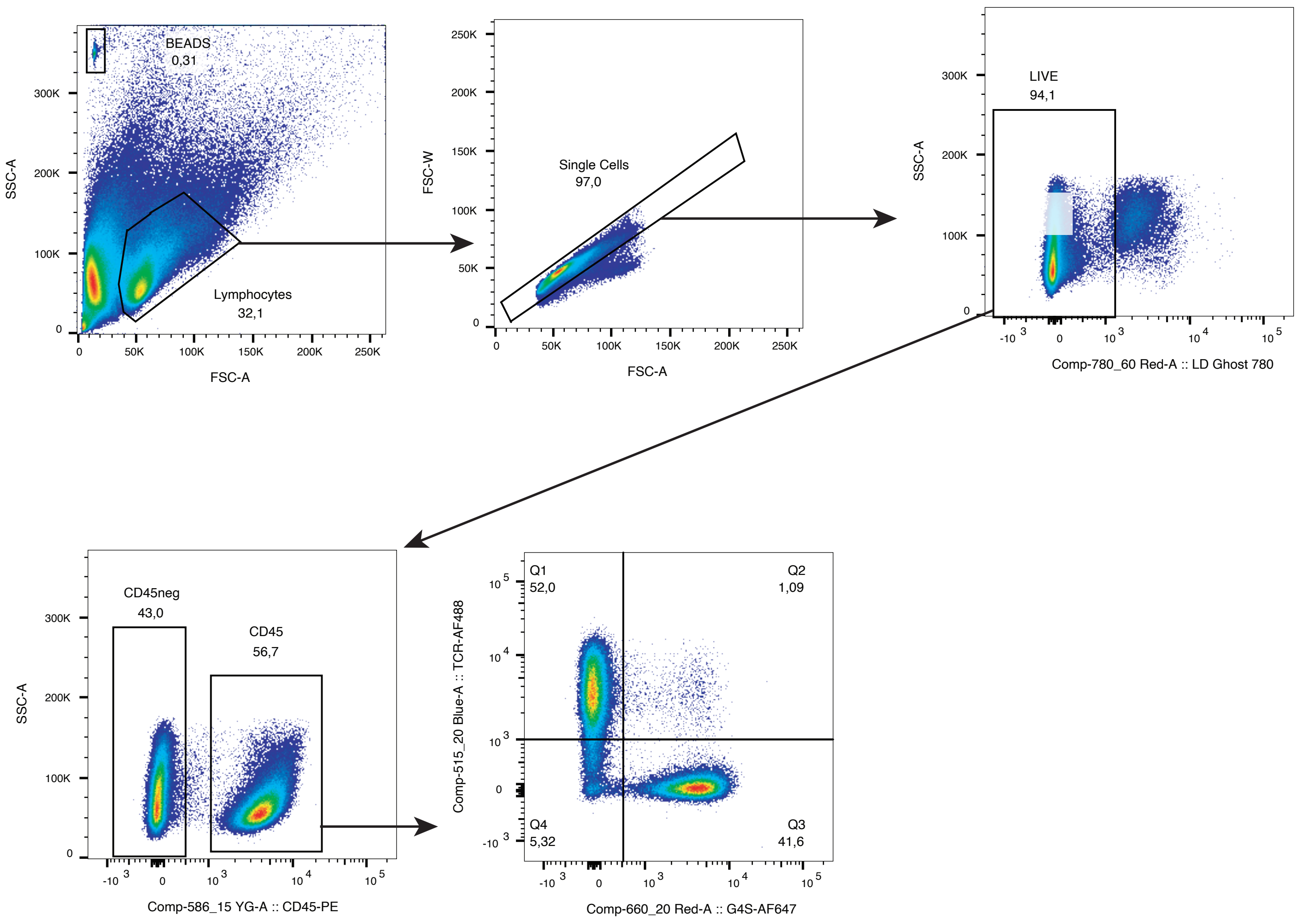

Supplementary Figure 2: Gating strategy for Figure 3b-d

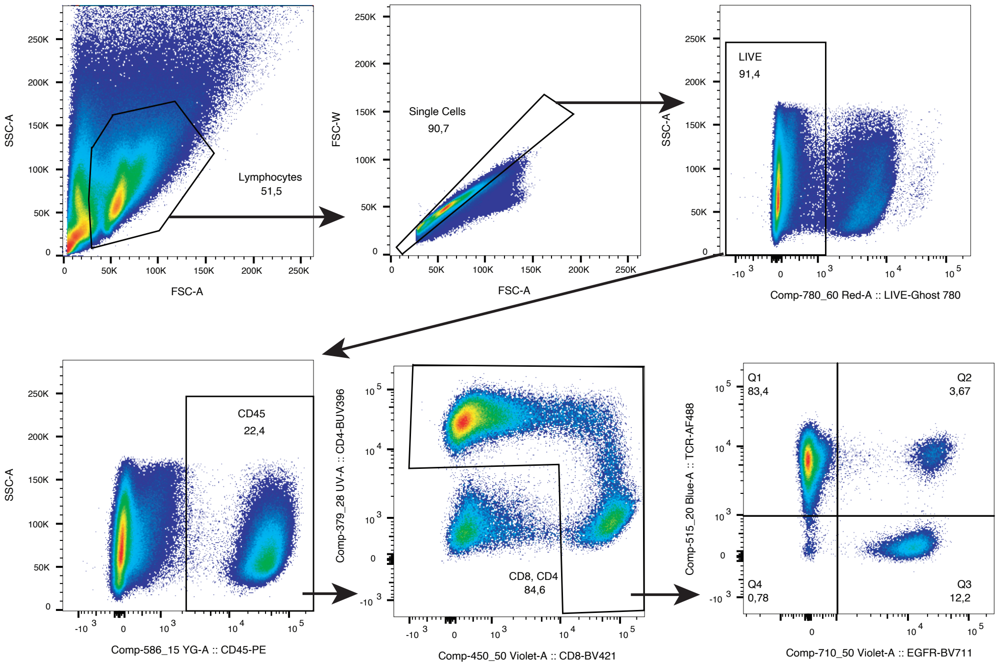

Gating strategy for Figure 3g-j

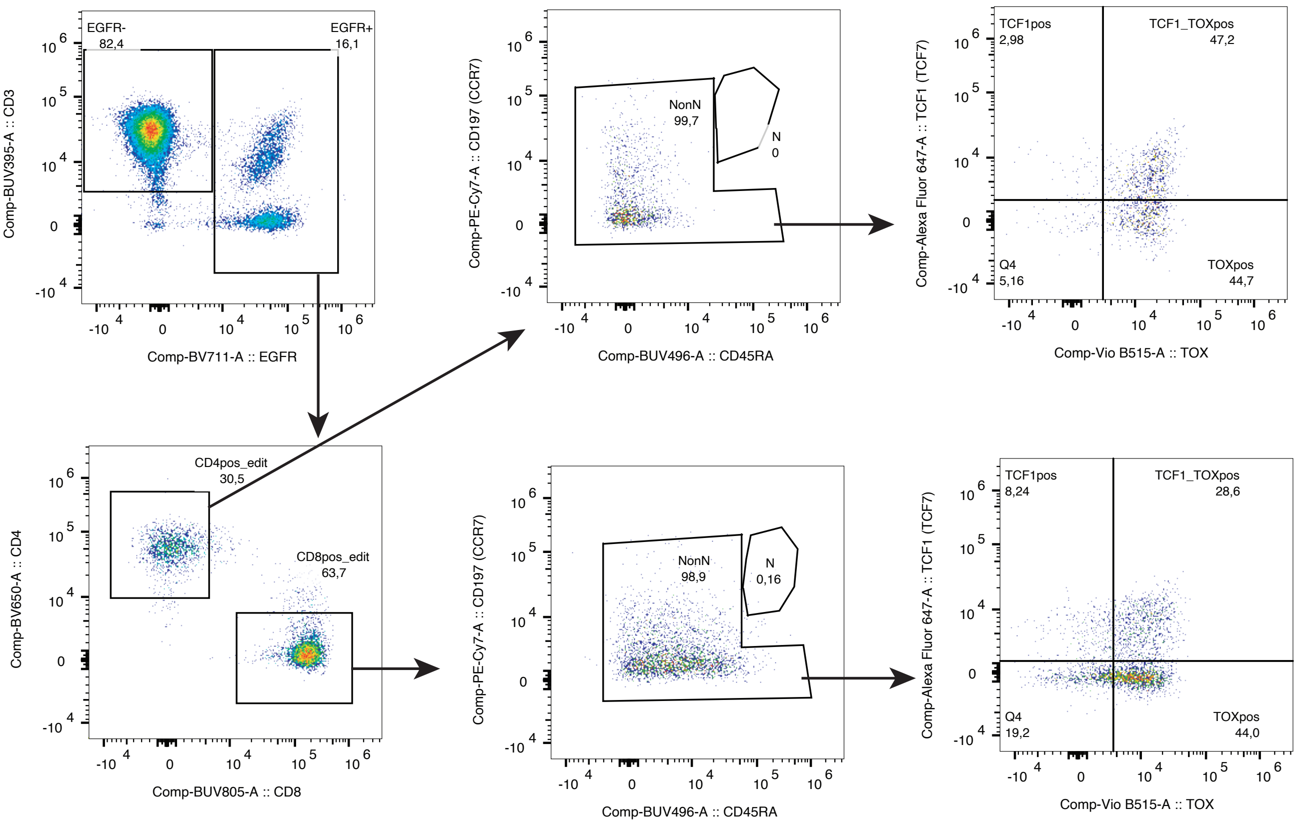

Supplementary Figure 3: Gating strategy for Figure 3 e,f,j and Extended Data 5c,d

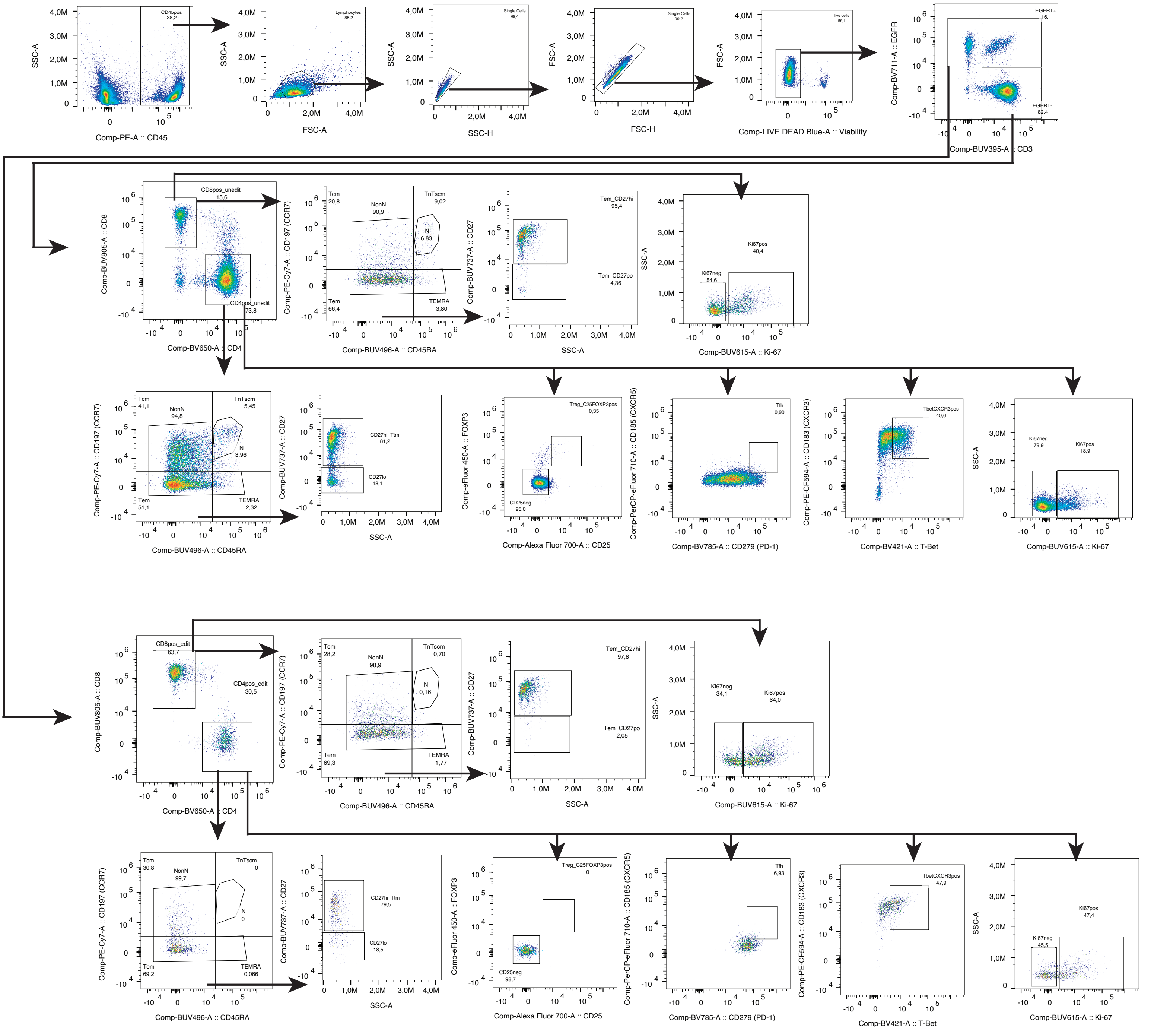

Supplementary Figure 4: Gating strategy for Figure 4g-j

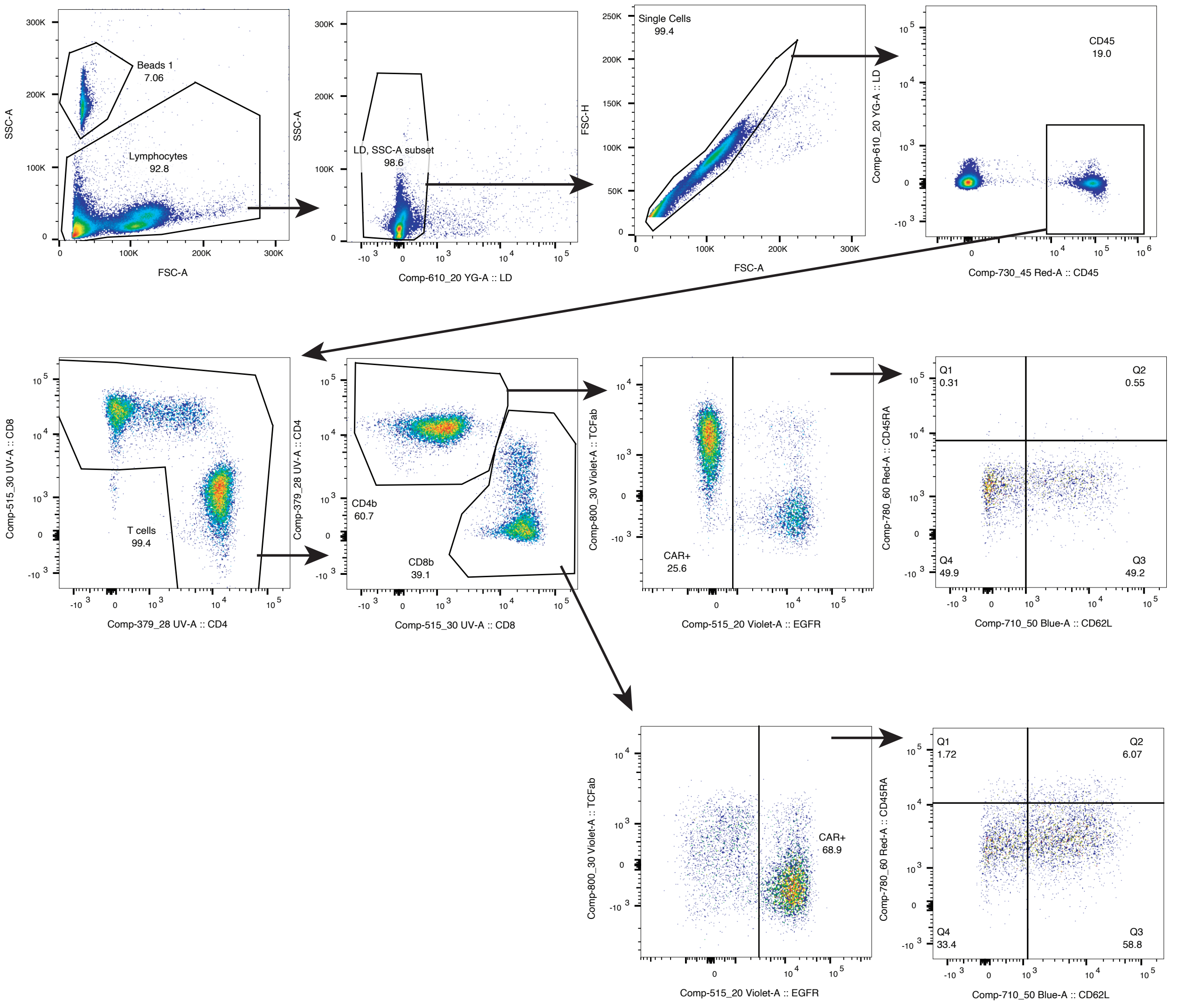

Supplementary Figure 5: Gating strategy for Extended Data 5a,b

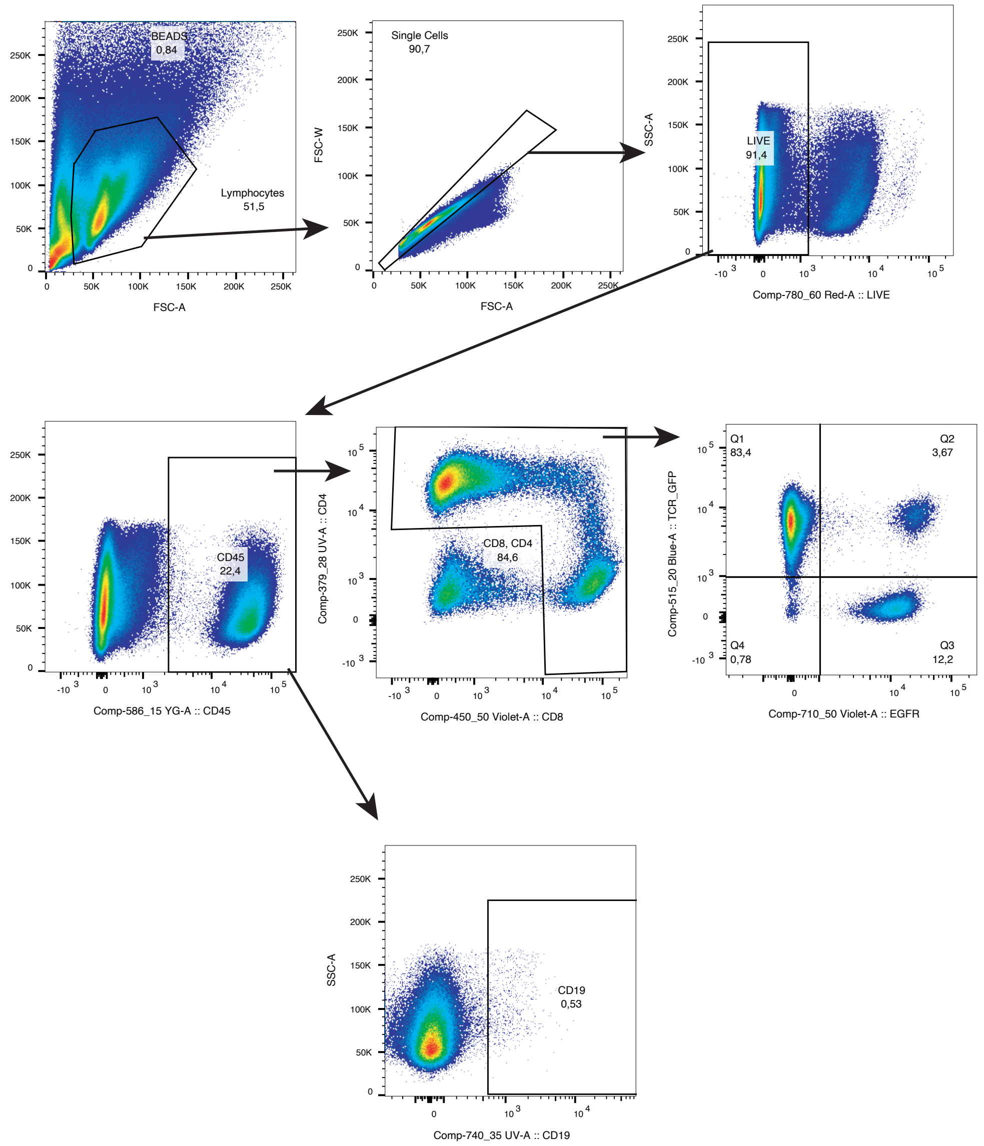

Supplementary Figure 6: Gating strategy for Extended Data 8d-i

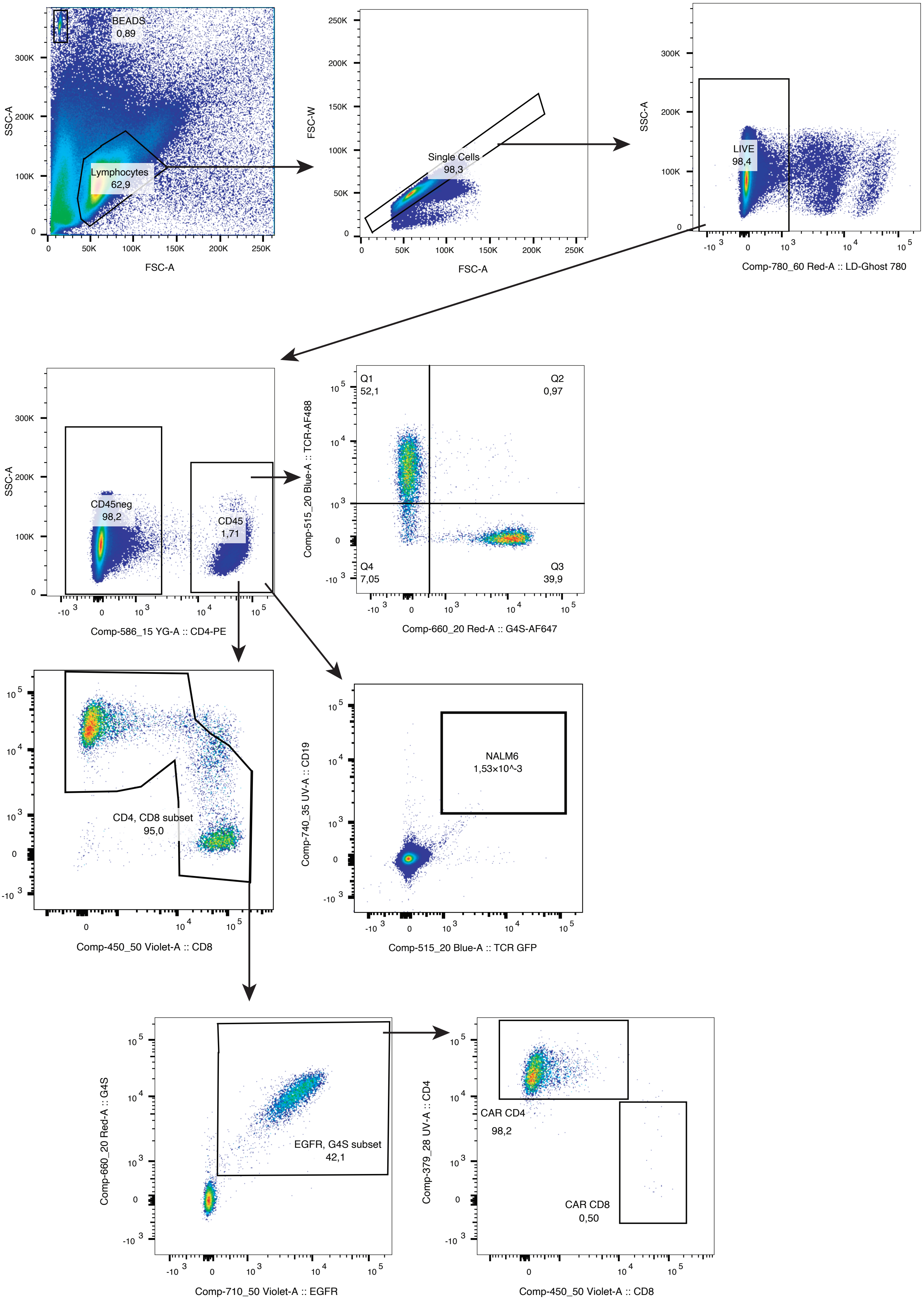

Supplement: Supplementary file 1 — Supplementary Figs. 1–6: gating strategies for the figures and extended data figures. [file 41586_2026_10235_MOESM1_ESM.pdf]
