# Supplementary material for: In vivo site-specific engineering to reprogram T cells
Source: Nature. 2026 Mar 18;652(8110):712–21. doi: 10.1038/s41586-026-10235-x (PMC13083257; doi:10.1038/s41586-026-10235-x)
Supplement: Supplementary file 2 — Reporting Summary [file 41586_2026_10235_MOESM2_ESM.pdf]

nature portfolio

Corresponding author(s): Justin Eyquem

Last updated by author(s): 2026/1/26

Reporting Summary

Nature Portfolio wishes to improve the reproducibility of the work that we publish. This form provides structure for consistency and transparency in reporting. For further information on Nature Portfolio policies, see our Editorial Policies and the Editorial Policy Checklist.

Statistics

For all statistical analyses, confirm that the following items are present in the figure legend, table legend, main text, or Methods section.

n/a	Confirmed	
☐	☒	The exact sample size (*n*) for each experimental group/condition, given as a discrete number and unit of measurement
☐	☒	A statement on whether measurements were taken from distinct samples or whether the same sample was measured repeatedly
☐	☒	The statistical test(s) used AND whether they are one- or two-sided<br>*Only common tests should be described solely by name; describe more complex techniques in the Methods section.*
☐	☒	A description of all covariates tested
☐	☒	A description of any assumptions or corrections, such as tests of normality and adjustment for multiple comparisons
☐	☒	A full description of the statistical parameters including central tendency (e.g. means) or other basic estimates (e.g. regression coefficient) AND variation (e.g. standard deviation) or associated estimates of uncertainty (e.g. confidence intervals)
☐	☒	For null hypothesis testing, the test statistic (e.g. *F*, *t*, *r*) with confidence intervals, effect sizes, degrees of freedom and *P* value noted<br>*Give P values as exact values whenever suitable.*
☒	☐	For Bayesian analysis, information on the choice of priors and Markov chain Monte Carlo settings
☒	☐	For hierarchical and complex designs, identification of the appropriate level for tests and full reporting of outcomes
☒	☐	Estimates of effect sizes (e.g. Cohen's *d*, Pearson's *r*), indicating how they were calculated

Our web collection on statistics for biologists contains articles on many of the points above.

Software and code

Policy information about availability of computer code

Data collection	Collection and analysis were performed using GraphPad Prism 10 (Dotmatics), FlowJo 10.10.0 (BD) and LivingImage Software.
Data analysis	Statistical analyses were performed using GraphPad Prism 10 (Dotmatics) and FlowJo 10.10.0 (BD).

For manuscripts utilizing custom algorithms or software that are central to the research but not yet described in published literature, software must be made available to editors and reviewers. We strongly encourage code deposition in a community repository (e.g. GitHub). See the Nature Portfolio guidelines for submitting code & software for further information.

Data

Policy information about availability of data

All manuscripts must include a data availability statement. This statement should provide the following information, where applicable:

- Accession codes, unique identifiers, or web links for publicly available datasets
- A description of any restrictions on data availability
- For clinical datasets or third party data, please ensure that the statement adheres to our policy

Data are available in the main text or the Supplementary Information. The data for the genome-wide screen described in this study have been deposited in the NCBI BioProject database under accession number PRJNA1412486.

Research involving human participants, their data, or biological material

Policy information about studies with human participants or human data. See also policy information about sex, gender (identity/presentation), and sexual orientation and race, ethnicity and racism.

Reporting on sex and gender	We used peripheral blood donated from healthy individuals in this study. We used both male and female donors.
Reporting on race, ethnicity, or other socially relevant groupings	We used peripheral blood donated from healthy individuals in this study. We used donors from multiple ethnic background to increase genetic diversity among donors
Population characteristics	We used healthy donors, and specifically request non-smoking and non-obese donors in this study
Recruitment	N/A
Ethics oversight	N/A

Note that full information on the approval of the study protocol must also be provided in the manuscript.

Field-specific reporting

Please select the one below that is the best fit for your research. If you are not sure, read the appropriate sections before making your selection.

☒ Life sciences ☐ Behavioural & social sciences ☐ Ecological, evolutionary & environmental sciences

For a reference copy of the document with all sections, see nature.com/documents/nr-reporting-summary-flat.pdf

Life sciences study design

All studies must disclose on these points even when the disclosure is negative.

Sample size	Sample size were chosen based on power calculation and pilot experiments. Due to the challenge in producing reagents in very large quantities, some experiments have unequal group sizes
Data exclusions	No data was excluded
Replication	All experiments were preceded with pilot experiments and replicated.
Randomization	Mouse groups were homogenized in weight, age, tumor or PBMC engraftment and Treatment groups were randomly assigned to mice.
Blinding	No blinding was done in this project.

Reporting for specific materials, systems and methods

We require information from authors about some types of materials, experimental systems and methods used in many studies. Here, indicate whether each material, system or method listed is relevant to your study. If you are not sure if a list item applies to your research, read the appropriate section before selecting a response.

Materials & experimental systems

n/a	Involved in the study	
☐	☒	Antibodies
☐	☒	Eukaryotic cell lines
☒	☐	Palaeontology and archaeology
☐	☒	Animals and other organisms
☒	☐	Clinical data
☒	☐	Dual use research of concern
☒	☐	Plants

Methods

n/a	Involved in the study	
☒	☐	ChIP-seq
☐	☒	Flow cytometry
☒	☐	MRI-based neuroimaging

Antibodies

Antibodies used	anti-CD7 PE-Cy7 (BioLegend #395610)<br><br>anti-CD7 APC (BioLegend #395606)

anti-EGFR BV510 (BioLegend #352938)

anti-TCRα/β BV786 (BD #563825)

anti-CD8 BUV496 (BD #612942)

anti-CD62L RB705 (BD #57094)

anti-CD45 R718 (BD #566961)

anti-CD45RA APC/Fire 750 (BioLegend #304152)
anti-TCRα/β AF488 (BioLegend #306712)

anti-CAS AF647 (Cell Signaling Technologies #6782)

anti-CD45 PE (BioLegend #368510)

anti-CD25 BV711 (BioLegend #356138)

anti-CD69 PE (BioLegend #310906)

anti-CD4 BV395 (BioLegend #563550)

anti-CD8 BV421 (BD #563217)

anti-CD8 BV711 (BioLegend #344734)

anti-EGFR AF488 (BioLegend #352908)

anti-EGFR BV711 (BioLegend #352290)

anti-CD3 BV737 (BD #612756)

anti-CD3 BV395 (BD #563546)

anti-CD56 BV711 (BioLegend #318336)

LIVE/DEAD Blue (ThermoFisher/Invitrogen L34961)

BV496 CD45RA (BD #741182)

BUV563 CD28 (BD Biosciences #741392)

BUV615 Ki67 (BD Biosciences #366-5609-80)

BUV737 CD27 (M-T271, BD #741833)

BUV805 CD8 SK1 (BD #612890)

BV421 T-bet 4B10 (BioLegend #644815)

Pacific Blue FOXP3 206D (BioLegend #320116)

BV510 CD19 HIB19 (BioLegend #302241)

BV605 CD39 A1 (BioLegend #328235)

BV650 CD4 OKT4 (BioLegend #317435)

BV711 EGFR AY13 (BioLegend #352290)

BV785 PD-1 EH12.2H7 (BioLegend #329930)

VioB515 TOX REA473 (Miltenyi #130-129-208)

PerCP-eFluor710 CXCR5 MU5UBEE (eBioscience #46-9185-41)

PE CD45 2D1 (BioLegend #368510)

PE-CF594 CXCR3 1C6 (BD #562451)

PE-Cy5 CD95 DX2 (BioLegend #305610)

PE-Cy7 CCR7 CD197 G043H7 (BioLegend #353225)

AF647 TCF7 7F11A10 (BioLegend #655204)

A700 CD25 BC96 (BioLegend #302621)

APC-F750 CD127 A019D5 (BioLegend #351349)

Validation	Each antibody was used following the manufacturer recommendations, and further optimized and validated in our large phenotyping panels using compensation controls such as FMOs and single antibody staining.

Eukaryotic cell lines

Policy information about cell lines and Sex and Gender in Research

Cell line source(s)	HEK293T (ATCC);<br>NALM-6 (provided by Michel Sadelain, Memorial Sloan Kettering Cancer Center);<br>JeKo-1 (provided by Arun Wiita, UCSF);<br>Raji (ATCC);<br>SupB15 (ATCC);<br>MES-SA (provided by the Roybal Lab, UCSF);<br>OPM-2 (ATCC)
Authentication	All the lines were authenticated either directly obtained and authenticated by ATCC, or authenticated by the collaborators who shared them.
Mycoplasma contamination	All cell lines have tested negative for mycoplasma.
Commonly misidentified lines<br>(See ICLAC register)	*Name any commonly misidentified cell lines used in the study and provide a rationale for their use.*

Animals and other research organisms

Policy information about studies involving animals; ARRIVE guidelines recommended for reporting animal research, and Sex and Gender in Research

Laboratory animals	NSG (NOD.Cg-Prkdc^scid Il2rg^tm1Wjl/SzJ; The Jackson Laboratory, JAX #005557);<br>NSG-MHC I/II double knockout (The Jackson Laboratory, JAX #025216);<br>hu-PBMC-NSG (The Jackson Laboratory, JAX #745557). All mice were injected and included in experiments at 8-10 weeks of age.
Wild animals	N/A
Reporting on sex	Each experiment in this study used either male or female mice. The experiments were mixed between male and female. Due to availability, more female mice were used.
Field-collected samples	N/A
Ethics oversight	All mice in the study were treated following a protocol approved by the UCSF Institutional Animal Care and Use Committee (IACUC), protocol number AN182757.

Note that full information on the approval of the study protocol must also be provided in the manuscript.

Plants

Seed stocks	N/A
Novel plant genotypes	N/A
Authentication	N/A

Flow Cytometry

Plots

Confirm that:

☒ The axis labels state the marker and fluorochrome used (e.g. CD4-FITC).

☒ The axis scales are clearly visible. Include numbers along axes only for bottom left plot of group (a 'group' is an analysis of identical markers).

☒ All plots are contour plots with outliers or pseudocolor plots.

☒ A numerical value for number of cells or percentage (with statistics) is provided.

Methodology

Sample preparation	Cells were stained in 100uL FACS buffer (2% FBS and 1mM EDTA in PBS) for 30 min at room temperature
Instrument	Fortessa LSR X50 (BD), Aurora spectral flow cytometer (Cytek Biosciences)
Software	SpectroFlo v3.3 software (Cytek Biosciences), FlowJo v10.10 (BD Biosciences), FACSDiva v9.0 (BD Biosciences)
Cell population abundance	The percentage of each population is provided in the figure, text, legend or supplementary data.
Gating strategy	The gating strategy is described in the text, legends or methods for all experiments.

☒ Tick this box to confirm that a figure exemplifying the gating strategy is provided in the Supplementary Information.
